# Temporal trends in population attributable fractions of modifiable risk factors for dementia: a time-series study of the English Longitudinal Study of Ageing (2004–2019)

**DOI:** 10.1186/s12916-024-03464-2

**Published:** 2024-06-26

**Authors:** Shanquan Chen, Benjamin R. Underwood, Rudolf N. Cardinal, Xi Chen, Shu Chen, Jay Amin, Huajie Jin, Jing Huang, Christoph Mueller, Lijing L. Yan, Carol Brayne, Hannah Kuper

**Affiliations:** 1 International Centre for Evidence in Disability, London, School of Hygiene & Tropical Medicine , London, WC1E 7HT UK; 2 Department of Psychiatry, University of Cambridge, Cambridge, CB2 0SZ UK; 3 Cambridgeshire and Peterborough NHS Foundation Trust, Cambridge, CB21 5EF UK; 4 School of Public Health, Yale University, New Haven, CT USA; 5 The ARC Centre of Excellence in Population Ageing Research (CEPAR), School of Risk and Actuarial Studies, University of New South Wales, Sydney, Australia; 6 Clinical Neurosciences, Faculty of Medicine, University of Southampton, Southampton, UK; 7 Southern Health NHS Foundation Trust, Southampton, UK; 8 King’s Health Economics, Institute of Psychiatry, Psychology & Neuroscience at King’s College London, London, UK; 9 Department of Occupational and Environmental Health Sciences, Peking University School of Public Health, Beijing, China; 10 Institute for Global Health and Development, Peking University , Beijing, China; 11 King’s College London, London, UK; 12 South London and Maudsley NHS Foundation Trust, London, UK; 13 Global Health Research Center, Duke Kunshan University, Jiangsu, China; 14 School of Public Health, Wuhan University, Wuhan, China; 15 Duke Global Health Institute, Duke University, Durham, NC USA; 16 Department of Preventive Medicine, Feinberg School of Medicine, Northwestern University, Chicago, IL USA; 17 Institute of Public Health, Forvie Site, University of Cambridge, Cambridge, CB2 2SR UK

**Keywords:** Temporal trend, Disparity, Population attributable fractions, Dementia, England

## Abstract

**Background:**

Interest in modifiable risk factors (MRFs) for dementia is high, given the personal, social, and economic impact of the disorder, especially in ageing societies such as the United Kingdom. Exploring the population attributable fraction (PAF) of dementia attributable to MRFs and how this may have changed over time remains unclear. Unravelling the temporal dynamics of MRFs is crucial for informing the development of evidence-based and effective public health policies. This investigation examined the temporal trajectories of MRFs for dementia in England.

**Methods:**

We used data from the English Longitudinal Study of Ageing, a panel study over eight waves collected between 2004 and 2019 (76,904 interviews in total). We calculated the PAFs for twelve MRFs (including six early- to mid-life factors and six late-life factors), as recommended by the Lancet Commission, and the individual weighted PAFs (IW-PAFs) for each risk factor. Temporal trends were analysed to understand the changes in the overall PAF and IW-PAF over the study period. Subgroup analyses were conducted by sex and socioeconomic status (SES).

**Results:**

The overall PAF for dementia MRFs changed from 46.73% in 2004/2005 to 36.79% in 2018/2019, though this trend was not statistically significant. During 2004–2019, hypertension, with an average IW-PAF of 8.21%, was the primary modifiable determinant of dementia, followed by obesity (6.16%), social isolation (5.61%), hearing loss (4.81%), depression (4.72%), low education (4.63%), physical inactivity (3.26%), diabetes mellitus (2.49%), smoking (2.0%), excessive alcohol consumption (1.16%), air pollution (0.42%), and traumatic brain injury (TBI) (0.26%). During 2004–2019, only IW-PAFs of low education, social isolation, and smoking showed significant decreasing trends, while IW-PAFs of other factors either did not change significantly or increased (including TBI, diabetes mellitus, and air pollution). Upon sex-specific disaggregation, a higher overall PAF for MRFs was found among women, predominantly associated with later-life risk factors, most notably social isolation, depression, and physical inactivity. Additionally, hearing loss, classified as an early- to mid-life factor, played a supplementary role in the identified sex disparity. A comparable discrepancy was evident upon PAF evaluation by SES, with lower income groups experiencing a higher dementia risk, largely tied to later-life factors such as social isolation, physical inactivity, depression, and smoking. Early- to mid-life factors, in particular, low education and obesity, were also observed to contribute to the SES-associated divergence in dementia risk. Temporal PAF and IW-PAF trends, stratified by sex and SES, revealed that MRF PAF gaps across sex or SES categories have persisted or increased.

**Conclusions:**

In England, there was little change over time in the proportion of dementia attributable to known modifiable risk factors. The observed trends underscore the continuing relevance of these risk factors and the need for targeted public health strategies to address them.

**Supplementary Information:**

The online version contains supplementary material available at 10.1186/s12916-024-03464-2.

## Background

Dementia syndrome is the second leading cause of disability-adjusted life years (DALY) in the United Kingdom (UK) [1]. The impact of dementia is projected to intensify, with the number of individuals affected expected to increase from approximately 0.89 million in 2019 to nearly 1.6 million by 2040 due to population ageing [2]. Despite extensive research, no disease-modifying drugs are approved for use in the UK, which underscores the necessity of targeting modifiable risk factors (MRFs) as a preventive strategy [3]. Given the projected increase in the numbers affected, a 5% reduction in incidence could potentially translate into a societal saving of around 3.7 billion pounds by 2040 [4, 5].

The population attributable fraction (PAF) is a common metric in disease prevention research, used to quantify the proportion of cases that could be averted, assuming causality, if specific risk factor(s) were to be eradicated [6–9]. The Lancet Commission on Dementia Prevention, Intervention, and Care (“the Lancet Commission”) used this metric and estimated that 40% of dementia cases globally could be attributable to 12 modifiable risk factors. These include one early-life factor (low education), five mid-life factors (hearing loss, hypertension, obesity, excessive alcohol consumption, and traumatic brain injury [TBI]), and six later-life factors (smoking, depression, physical inactivity, social isolation, diabetes mellitus, and air pollution) [10]. The MRF PAF for dementia has been estimated for various countries (including Australia [9, 11], US [6, 12], Greek [12], New Zealand [8], Canada [13], India [7], China [7, 14–16], Chile [17], several Latin American countries [7], Italy [18], Japan [19], and Sweden [20]). These studies vary greatly in their estimated PAF for MRFs, underscoring the necessity for country-specific evaluations and likely intervention strategies. Moreover, a decline in dementia incidence has been noted in some countries [21, 22], illustrating the potential practical contribution of focusing on the PAF in dementia research and policy making.

The UK government has been proactive in dementia prevention initiatives, as exemplified by charters such as “Challenge of Dementia 2012–2015” and “Challenge of Dementia 2020,” as well as the recent guidance “Dementia: Applying All Our Health” issued by the Office for Health Improvement and Disparities (OHID) in October 2021. However, the most recent UK-specific PAF estimation for dementia, which only considered seven modifiable risk factors, was based on data from 2006 [23] and 2014 [10]. Given the identification of new dementia risk factors and significant epidemiological shifts in previously known risk factors [10, 23, 24], there is an urgent need for updated estimations.

Over the past few decades, UK has witnessed notable changes in the prevalence of several key dementia risk factors, such as increases in obesity [25] and diabetes mellitus [26], and decreases in smoking [27] and hypertension [28]. These shifts can substantially impact the contribution of each factor to the overall dementia risk, but their relative importance on dementia PAF has not been thoroughly investigated in England. Understanding these temporal shifts is crucial for guiding public health policies and interventions aimed at reducing the burden of dementia. Therefore, temporal trends in MRF PAF for dementia need to be evaluated to assess progress, identify areas of concern, and inform future preventive strategies.

Another notable evidence gap in the UK is the lack of PAF estimations for dementia stratified by sex and socioeconomic status (SES), despite well-documented disparities in dementia incidence across these groups [29, 30]. Identifying the key factors driving dementia within different sex or socioeconomic groups is a critical step towards devising equitable and precisely targeted health policies.

Approximately 85% of individuals with dementia in the UK reside in England [4, 5]. This study aims to address the evidence gaps described above, using longitudinal data from England spanning the years 2004–2019. The study aims to answer four primary questions: (1) What proportion of dementia cases in England is attributable to potentially MRFs? (2) Which MRFs are the primary drivers of the dementia burden in England? (3) What are the temporal trends in these MRF PAFs? (4) Do these results differ by sex and socioeconomic status?

## Methods

### Data source and participants

This study used publicly available data derived from the English Longitudinal Study of Ageing (ELSA) in the UK [31]. ELSA is a biennial longitudinal study, initiated in 2002, designed to represent individuals aged 50 and above residing in private households within England. The original ELSA sample was drawn from respondents to the Health Survey for England (HSE), an annual cross-sectional survey, in the years 1998, 1999, and 2001 [32]. ELSA employs a multi-stage probability sampling design to ensure national representativeness. The sampling frame covers all private households in England containing at least one individual aged 50 or older [32]. To maintain representativeness over time, ELSA employs several strategies. These include follow-up surveys with rigorous tracking methods to minimize attrition, the use of weighting to adjust for any non-response or sampling biases, and periodically refreshing its cohort in each wave to maintain age representation [32]. The weights in ELSA are calculated through a multi-step process that accounts for the differential probabilities of selection, non-response, calibration to known population totals, and trimming and scaling to reduce the influence of extreme values [32]. In each wave, the same standardized protocol was used, ensuring consistency in measures and sampling methods. Comparisons with the UK Census data show that ELSA is largely representative of the English population aged 50 + in terms of age, gender, and geographical distribution. For instance, in the 2011 census, 49.7% of females aged 50 or above in England were between 50 and 64 years old, while the weighted percentage for the same age group in the ELSA wave 5 (2010–2011) samples was 48.5%. Similarly, the 2021 census showed that 48.8% of individuals aged 50 or above were in the 50–64 age group, compared to the weighted percentage of 52.6% in the ELSA wave 9 (2018–2019) samples. Trained personnel conducted Computer-Assisted Personal Interviews (CAPI) within the participants' homes. For those unable or unwilling to participate personally, proxy respondents, generally a spouse or other family member, were used. Such proxy interviews accounted for approximately 2% of all interviews [32]. ELSA encompasses a comprehensive set of data, including sociodemographic attributes, health status and diagnosis, health behaviors, and social networks. Additionally, health examination data and blood samples were collected through nurse visits every four years. The high quality of the ELSA dataset has enabled a recent study to successfully project the number of people with dementia in England up to 2040 [33]. Detailed explications of the data, sampling methodologies, and quality control procedures have been previously documented [32].

For this analysis, we incorporated data from waves 2 to 9 of ELSA, covering the period from 2004–05 to 2018–19, as nurse visit data became available starting from wave 2. There were a total of 78,038 in-person interviews (ranging from 8,475 to 11,050 for each wave), with an approximate retention rate of 80% [34]. We excluded 1,134 in-person interviews if that person had a prior diagnosis of dementia or memory-related disorders, determined by the self-reported question, “Has a doctor ever told you that you had/currently have Alzheimer’s disease, dementia, organic brain syndrome, senility, or any other serious memory impairment.” Among the remaining cases, there were missing values as follows: SES missing in 1.8%, depression scores 4.3%, air pollution 13.5%, body height 19%, body weight 23.3%, and alcoholic drink days 24.2%. Missing values were imputed for primary analysis or omitted for a sensitivity analysis (further details provided in the data analysis section).

### Modifiable risk factors

This study incorporated all 12 factors proposed by the Lancet Commission for the assessment of dementia risk factors. An extensive rationale for the selection of these 12 factors is provided in the Lancet Commission publications [10, 24]. Definitions of each risk factor are outlined in Table 1. Where feasible, we adhered to the risk factor definitions used in the Lancet Commission publications. However, in cases where the ELSA data did not provide corresponding information, alternative definitions were adopted from high-quality studies [6–9, 20], which similarly computed the PAF based on the Lancet Commission's propositions.

**Table 1:** Definitions of dementia risk factors

Risk factors	Definition in the report of the Lancet Commission	Definition in this study
**Early-life (age < 45 years)**		
Low education (< = 65)	None or primary school only; or no secondary school education; or formal education to a maximum age of 11–12 years	Self-reported highest education attained is less than high school
**Midlife (age 45–64 years)**		
Hearing loss	Hearing loss present at a threshold of 25 dB, which is the World Health Organization threshold for hearing loss	Meet at least one of the following criteria: 1. Self-rated hearing ability as poor, regardless of the use of hearing aids; 2. Investigator believes the respondent has a hearing impairment, measured by the question "what factors may have impaired the respondent's performance", with the response of deaf or hard of hearing, or by the question "please indicate whether any of the following problems occurred in relation to word recall", with the response that respondent had difficulty hearing any of the words
Hypertension	Systolic BP of 130 mm Hg or higher	Hypertension from blood pressure (i.e., systolic ≥ 130 mmHg or diastolic ≥ 90 mmHg) or self-reported diagnosis of hypertension
Obesity	Body mass index (BMI) ≥ 30	BMI ≥ 30
Excessive alcohol	Drinking more than 21 units (168 g) of alcohol weekly	Self-report of drinking more than 14 alcoholic drinks per week (average more than 2 drinks each day), based on the definition of alcohol misuse published by the NHS in the UK
Traumatic brain injury (TBI)	The International Classification of Disease (ICD) defines mild TBI as concussion and severe TBI as skull fracture, oedema, brain injury or bleed	People diagnosed with ICD-10 codes S02, S04, S06, S07, S09, T04.0, and T06.0
**Later-life (age ≥ 65 years)**		
Smoking	No definition provided	Self-reported by the affirmative response to "Do you currently smoke cigarettes?"
Depression	No definition provided	Score of Center for Epidemiologic Studies Depression Scale (CESD-8) > = 4, or self-reported diagnosis of depression
Physical inactivity	No definition provided. The report summarised that at least weekly midlife moderate-to-vigorous physical activity (breaking into a sweat) was associated with reduced dementia risk over a 25-year period of follow-up, and that more than 2.5 h of self-reported moderate-to-vigorous physical activity per week lowered dementia risk over 10 years	Self-report of “hardly ever” or “never” on both the following four-point Likert scales of level of physical activity (categories were “more than once a week”, “once a week”, “one to three times a month”, “hardly ever”, or “never”): 1. Do you take part in sports or activities that are vigorous? 2. Do you take part in sports or activities that are moderate?
Social isolation	Social isolation was not measured directly. Instead, living alone (i.e., no cohabitation) was used as a proxy measure	Living alone, no other people living in the household
Diabetes mellitus	No definition provided	HbA1c ≥ 6.5% or self-reported diabetes mellitus
Air pollution	No definition provided. The report summarised that high nitrogen dioxide (NO_2_) concentration (> 41·5 µg/m^3^; adjusted HR 1·2, 95% CI 1·0–1·3), fine ambient particulate matter (PM)2·5 from traffic exhaust (1·1, 1·0–1·2) and PM2·5 from residential wood burning (HR = 1·6, 95% CI 1·0–2·4 for a 1 μg/m^3^ increase) are associated with increased dementia incidence	Coded as "yes" if respondents used coal, oil, or wood in their home, either for heating or for any other purpose

Sex was determined by self-reported sex at birth, categorized as male or female. SES was approximated at each wave using non-housing financial wealth, which included earnings, savings, benefits, stocks, bonds, and gilts [35]. Housing wealth was excluded as it may not accurately reflect a household's current financial status or access to resources, and can be influenced by factors such as regional property value variations [36]. The comprehensive financial data collected in the ELSA dataset enabled the construction of a consistent and reliable measure of non-housing financial wealth across the study period. For our analysis, SES was categorized into quintiles based on the distribution of non-housing financial wealth.

### Calculation of population attributable fractions

The methodology employed for the computation of the population attributable fraction (PAF) is well-documented in existing literature [7, 10, 11, 23] and is provided here for clarity. The derivation of PAF necessitates three integral components: the prevalence of risk factors, the magnitude of the association between these risk factors and dementia (quantified by relative risk, RR), and the shared variance among these risks (referred to as 'communality'). PAF, expressed as a percentage (PAF%).

1 $${Individual\;PAF}_{i}=\frac{{prevalence}_{i}\times \left({RR}_{i}-1\right)}{1+{prevalence}_{i}\times \left({RR}_{i}-1\right)},i=\text{1,2}\dots 12$$

in which $i$ indicates corresponding parameters or estimates for risk factor $i$.

2 $$Overall\;PAF=1-\left(1-{weight}_{1}\times {PAF}_{1}\right)\times \left(1-{weight}_{2}\times {PAF}_{2}\right)\dots \left(1-{weight}_{12}\times {PAF}_{12}\right)$$

in which

3 $${Weight}_{i}=1-{communality}_{i}$$

4 $${Individual\;weighted\;PAF}_{i}=\frac{{Individual\;PAF}_{i}}{{\sum\limits}_{1}^{12}{Individual\;PAF}_{i}}\times Overall\;PAF$$

Individual PAF represents the proportion of dementia cases attributable to a specific risk factor, considering its prevalence and relative risk. Overall PAF, on the other hand, estimates the proportion of dementia cases attributable to the combined effect of all 12 risk factors, accounting for their overlap or shared variance between risk factors, known as communality. The communality is used to calculate weights for each risk factor, ensuring that the combined effect of all risk factors does not exceed 100%. Individual weighted PAF represents the contribution of each risk factor to the overall PAF, this allows for a direct comparison of the relative importance of each risk factor in contributing to the overall dementia burden.

### Modifiable risk factor prevalence

It is noteworthy that certain factors, such as obesity and blood pressure, which are risk factors during middle age, have been shown to decrease before the onset of dementia due to the disease's progression [10]. Therefore, the Lancet Commission [10] has suggested that risk factors should be considered within specific periods of a person's life. In our study, we estimated the prevalence of the targeted MRFs (excluding TBI) among the ELSA participants within the age range suggested by the Lancet Commission [10]. However, we did not impose an age range restriction for low education, as ELSA does not include individuals younger than 45, and due to the reasonable assumption that low education levels in adults are unlikely to change with disease progression. Moreover, the Lancet Commission acknowledges that while they have identified particular age ranges for risk factors, these factors may also be pertinent outside of these specified periods [10, 24]. Following practices observed in certain high-quality publications [7, 8], individual PAF calculation for low education were not strictly confined to the specific age groups proposed by the Lancet Commission.

The prevalence of TBI, within the age range suggested by the Lancet Commission, was approximated from the incident rate of hospital admissions for head injury, using a transformation formula recommended by the US Centers for Disease Control and Prevention [37] (also available in Supplementary). The incidence rate of admission for head injury was extracted from a report based on the Hospital Episode Statistics system [38].

### Estimates of RR

The estimates for relative risk (RR) of the association of the individual MRFs with dementia incidence were derived from the 2020 Lancet Commission publication (also available in Supplementary Table 1) [10]. This publication employed systematic reviews and meta-analyses to procure RR estimates for the twelve risk factors. In the context of this research, it was posited that the RRs corresponding to the above twelve MRFs associated with dementia remain constant. This hypothesis is grounded in the findings from the Cognitive Function and Ageing Study conducted in the United Kingdom, which demonstrated the stability of risk structures over a temporal continuum [39].

### Communality

It is common for an individual to present with multiple risk factors concurrently (e.g., hypertension and diabetes). Thus, the PAF computed solely based on the prevalence of MRFs and their respective RRs in relation to dementia requires adjustment for communality (overlap between risk factors, as seen in formulas 2 and 3). Communality quantifies the proportion of shared variance among risk factors [23]. A risk factor with higher communality contributes to a smaller individual PAF. In this study, communality was calculated in accordance with methodologies used in prior dementia PAF studies [7, 10, 11, 23]: the computation of the tetrachoric correlation matrix between all risk factors, followed by a principal components analysis on that correlation matrix. Subsequently, the communality for each risk factor was determined as the sum of squares of the loadings in all principal components with an eigenvector greater than 1.

ELSA does not provide data on TBI. In line with the Lancet Commission's approach, the communality of TBI was represented by the mean of the communalities of the other 11 risk factors [10].

### Data analysis

All analyses were performed using the statistical software R project (version 4.3.0). We used the sampling weights provided by ELSA from each wave to generate representative estimations. We report two-tailed *p* values. *P* < 0 0.05 was considered to be statistically significant.

Missing data were imputed. We employed multiple imputations with chained equations, generating 25 imputed datasets to minimize bias and preserve statistical power [40].

We used Monte Carlo simulations (*n* = 1 000 000) to include uncertainty around the PAF estimation, according to distributional assumptions (beta distributions for prevalence and normal distributions for the logarithm of relative risks) [17]. The mean and the 2.5% and 97.5% quantiles obtained from PAF distributions were reported.

A series of linear regressions were fitted to test the temporal trend (average percentage change [APC]) per year of the PAF, with PAF as the outcome and the continuous form of year as the predictor.

We also repeated the analysis by sex or by SES. We calculated the between-group variance of PAF for each risk factor, to explore the contributions of risk factors to variance in PAF across sex or SES.

We performed two sensitivity analyses to estimate variability in PAF. First, we excluded cases with missing values (instead of imputing them) and repeated the analysis. Second, given the documented under-diagnosis of dementia [41, 42], we also excluded those with probable dementia or those where there was a proxy response during follow-up. Probable dementia cases were assessed based on a validated 25-point cognition scale, composed of immediate and delayed word recall tests (0–10 points, respectively) and self-rated memory (0–5 points) [33]. For each wave, individuals were classified as having probable dementia if their cognition scores were 1.5 standard deviations (SDs) below the population mean when stratified by education levels [33]. Once a person was identified as having potential dementia, the corresponding case was excluded from the analysis based on current and following waves.

## Results

### Description of the total data set

Across all the ELSA data for 2004–2019, there were a total of 76,904 in-person interviews, with an average age of 66.3 years (SD = 10.4). Most were women (55.7%), with one fifth in the upper SES quintile. During follow-up, hypertension, present in 59.2% of the participants, was the most common risk factor, followed by low levels of education (33.1%), measured obesity (31.2%), self-reported social isolation (23.2%), measured hearing loss (22.7%), air pollution (15.6%), self-reported physical inactivity (17.5%), depression (16.8%), excessive alcohol consumption (15.2%), smoking (12.7%), diabetes mellitus (11.3%), and traumatic brain injury (TBI, 0.9%). The basic description of the socio-demographic factors and 12 modifiable risk factors by survey year was presented in Table 2. In more recent ELSA waves, more sampled people were aged 65 or over (*p* < 0.001), but no significant difference in sex (*p* = 0.419) was observed. The weighted basic description was provided in Sup Table 2.

**Table 2:** Basic description of socio-demographic factors and 12 modifiable risk factors by survey year. Data presented as the number and percentage. The *p*-values were obtained using the Mantel–Haenszel Chi-squared test to assess the presence of significant trends in the prevalence of risk factors across the survey years

Variable	Year (= 2004–05) (*n* = 9365)	Year (= 2006–07) (*n* = 9671)	Year (= 2008–09) (*n* = 10,923)	Year (= 2010–11) (*n* = 10,122)	Year (= 2012–13) (*n* = 10,437)	Year (= 2014–15) (*n* = 9500)	Year (= 2016–17) *(n* = 8308)	Year (= 2018–19) (*n* = 8578)	*P*
**Socio-demographic factors**									
Age (> = 65)	4693 (50.1%)	4340 (44.9%)	5068 (46.4%)	5241 (51.8%)	5535 (53.0%)	5439 (57.3%)	5324 (64.1%)	5259 (61.3%)	< 0.001
Sex (= Female)	5286 (56.4%)	5430 (56.1%)	6058 (55.5%)	5620 (55.5%)	5767 (55.3%)	5275 (55.5%)	4631 (55.7%)	4804 (56.0%)	0.419
Wealth status									
Lowest	1882 (20.1%)	1935 (20.0%)	2168 (19.8%)	2020 (20.0%)	2145 (20.6%)	1886 (19.9%)	1661 (20.0%)	1704 (19.9%)	0.966
2	1867 (19.9%)	1907 (19.7%)	2206 (20.2%)	2017 (19.9%)	2051 (19.7%)	1895 (19.9%)	1654 (19.9%)	1721 (20.1%)	
3	1850 (19.8%)	1949 (20.2%)	2155 (19.7%)	2025 (20.0%)	2052 (19.7%)	1895 (19.9%)	1655 (19.9%)	1721 (20.1%)	
4	1892 (20.2%)	1958 (20.2%)	2206 (20.2%)	2039 (20.1%)	2103 (20.1%)	1911 (20.1%)	1666 (20.1%)	1726 (20.1%)	
Highest	1874 (20.0%)	1922 (19.9%)	2188 (20.0%)	2021 (20.0%)	2086 (20.0%)	1913 (20.1%)	1672 (20.1%)	1706 (19.9%)	
**Modifiable risk factors**									
Low education (= yes)	4021 (42.9%)	3658 (37.8%)	3880 (35.5%)	3451 (34.1%)	3275 (31.4%)	2765 (29.1%)	2312 (27.8%)	2074 (24.2%)	< 0.001
Hypertension (= yes)	5780 (61.7%)	5365 (55.5%)	6489 (59.4%)	6038 (59.7%)	6262 (60.0%)	5584 (58.8%)	5015 (60.4%)	4964 (57.9%)	0.595
Obesity (= yes)	2825 (30.2%)	2979 (30.8%)	3488 (31.9%)	3241 (32.0%)	3266 (31.3%)	3026 (31.9%)	2620 (31.5%)	2728 (31.8%)	0.026
Hearing loss (= yes)	2179 (23.3%)	2099 (21.7%)	2234 (20.5%)	2133 (21.1%)	2281 (21.9%)	2409 (25.4%)	2124 (25.6%)	2028 (23.6%)	< 0.001
Excessive alcohol (= yes)	1997 (21.3%)	2166 (22.4%)	1497 (13.7%)	1307 (12.9%)	1358 (13.0%)	1137 (12.0%)	1019 (12.3%)	1070 (12.5%)	< 0.001
Diabetes mellitus (= yes)	853 (9.1%)	883 (9.1%)	1230 (11.3%)	1225 (12.1%)	1160 (11.1%)	1121 (11.8%)	1074 (12.9%)	1119 (13.0%)	< 0.001
Social isolation (= yes)	2314 (24.7%)	2257 (23.3%)	2520 (23.1%)	2383 (23.5%)	2379 (22.8%)	2152 (22.7%)	1933 (23.3%)	1891 (22.0%)	< 0.001
Depression (= yes)	1538 (16.4%)	1749 (18.1%)	2004 (18.3%)	1919 (19.0%)	1866 (17.9%)	1667 (17.5%)	1119 (13.5%)	1182 (13.8%)	< 0.001
Physical inactivity (= yes)	1550 (16.6%)	1604 (16.6%)	1906 (17.4%)	1789 (17.7%)	1873 (17.9%)	1695 (17.8%)	1503 (18.1%)	1512 (17.6%)	0.001
Smoking (= yes)	1461 (15.6%)	1495 (15.5%)	1526 (14.0%)	1294 (12.8%)	1291 (12.4%)	1073 (11.3%)	782 (9.4%)	830 (9.7%)	< 0.001
Air pollution (= yes)	1263 (13.5%)	1336 (13.8%)	1583 (14.5%)	1573 (15.5%)	1692 (16.2%)	1642 (17.3%)	1389 (16.7%)	1514 (17.6%)	< 0.001
Traumatic brain injury (TBI) (= yes)a	0.6%	0.7%	0.8%	1.0%	0.9%	1.0%	0.9%	1.0%	< 0.001

^a^TBI was approximated from the incident rate of hospital admissions for head injury, using a transformation formula recommended by the US Centers for Disease Control and Prevention

### PAF trajectory in England

**Fig. 1:**
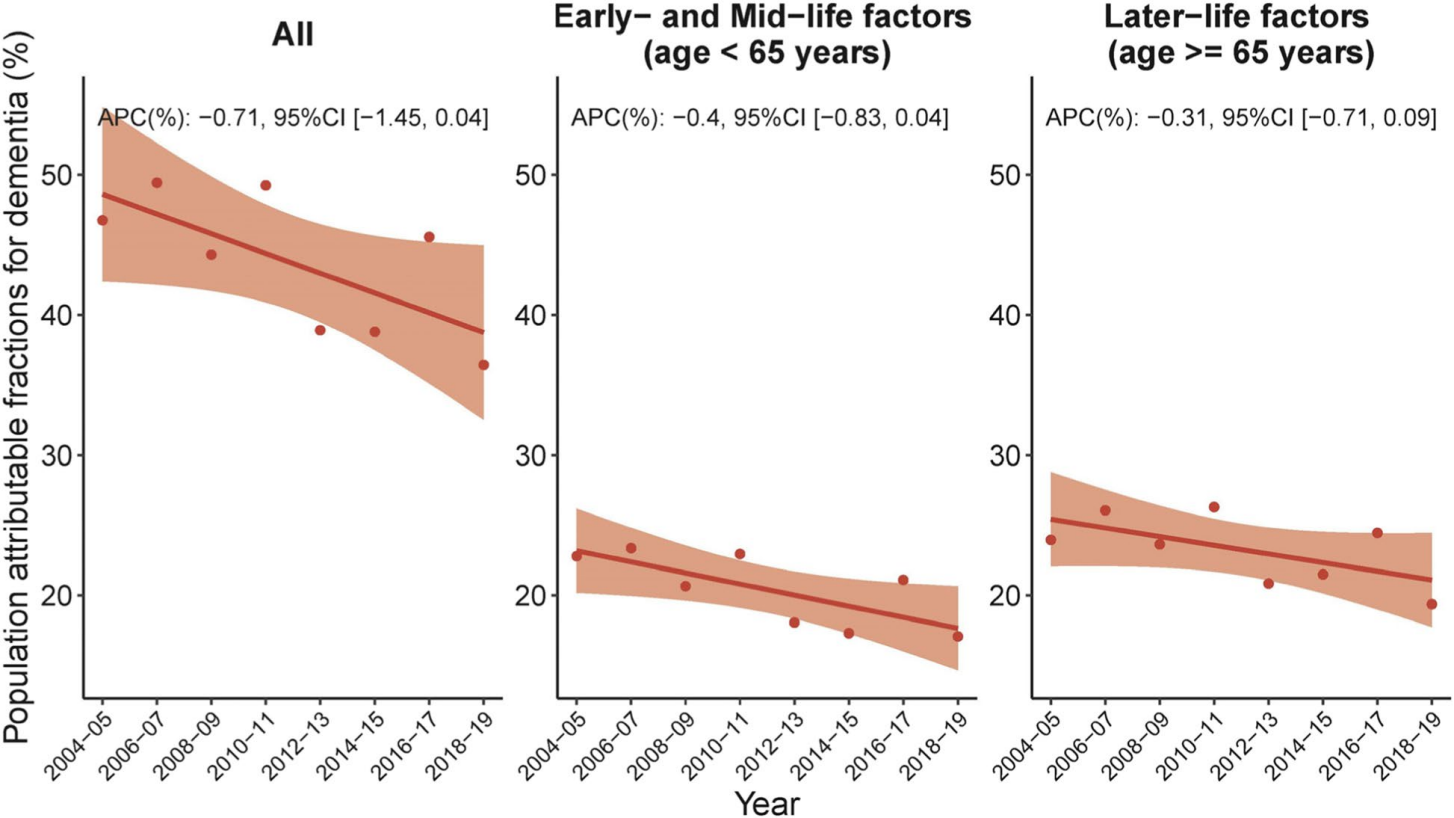
Overall temporal trends in population attributable fraction of 12 modifiable risk factors for dementia, 2004–2019. Average percentage change (APC) was used to quantify the temporal trend in population attributable fraction (PAF, as %), extracted from linear regression with PAF as the outcome and continuous form of year as the predictor. The APC indicates the extent to which the percentage points of PAF vary with each passing year

During 2004–2019, the overall MRF PAF for dementia decreased from 46.73% (95% confidence intervals [CI] [26.56,52.05]) in 2004/2005 to 36.79% (95% CI [20.40,51.44]) in 2018/2019 (Fig. 1, Panel C). However, this downward trend was not statistically significant (APC -0.71, 95% CI [-1.45, 0.04]). The overall MRF PAF was almost equally distributed between early- and mid-life factors and later-life factors, with the latter slightly greater (Fig. 1, Panel A and B). Over the study period, the PAF attributable to both early- and mid-life factors and later-life factors exhibited a non-significant decline trend.

**Fig. 2:**
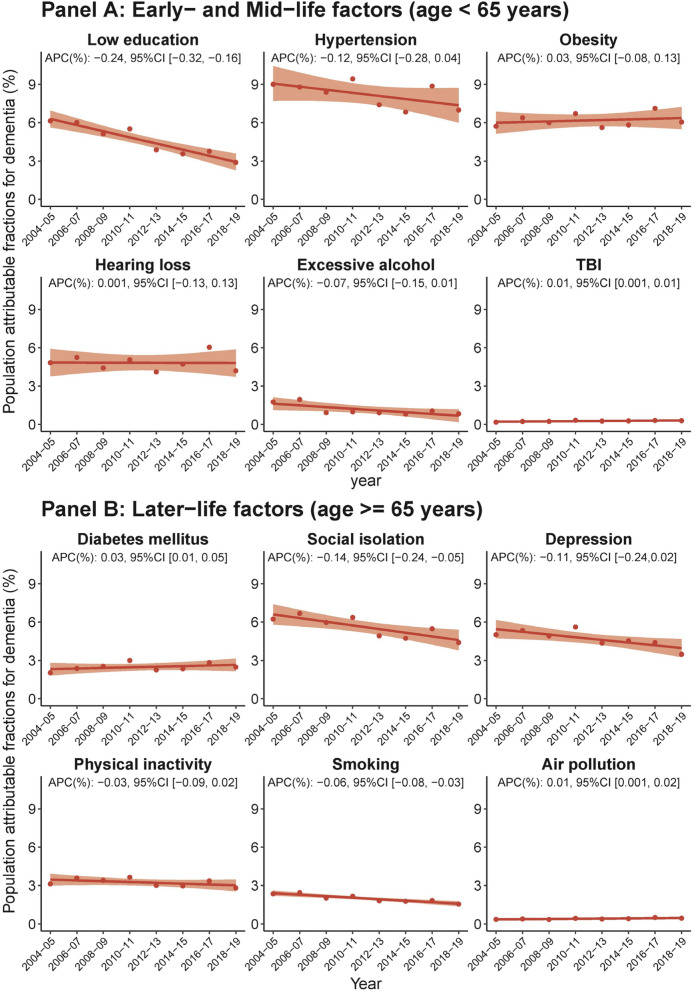
Temporal trends in population attributable fraction of 12 modifiable risk factors for dementia, 2004–2019, by risk factor. Average percentage change (APC) was used to quantify the temporal trend in population attributable fraction (PAF, as %), extracted from linear regression with PAF as the outcome and continuous form of year as the predictor. The APC indicates the extent to which the percentage points of PAF vary with each passing year

During 2004–2019, the individual weighted PAFs (IW-PAFs) for dementia by risk factors demonstrated that hypertension (8.21%) had the highest average IW-PAF, followed by obesity (6.16%), social isolation (5.61%), hearing loss (4.81%), depression (4.72%), low education (4.63%), physical inactivity (3.26%), diabetes mellitus (2.49%), smoking (2.0%), excessive alcohol consumption (1.16%), air pollution (0.42%), and TBI (0.26%). Notably, over the 15-year period studied, the IW-PAFs of low education, social isolation, and smoking showed significant decreasing trends, while TBI, diabetes mellitus, and air pollution displayed significant increasing trends (Fig. 2).

### PAF trajectory by sex

**Fig. 3:**
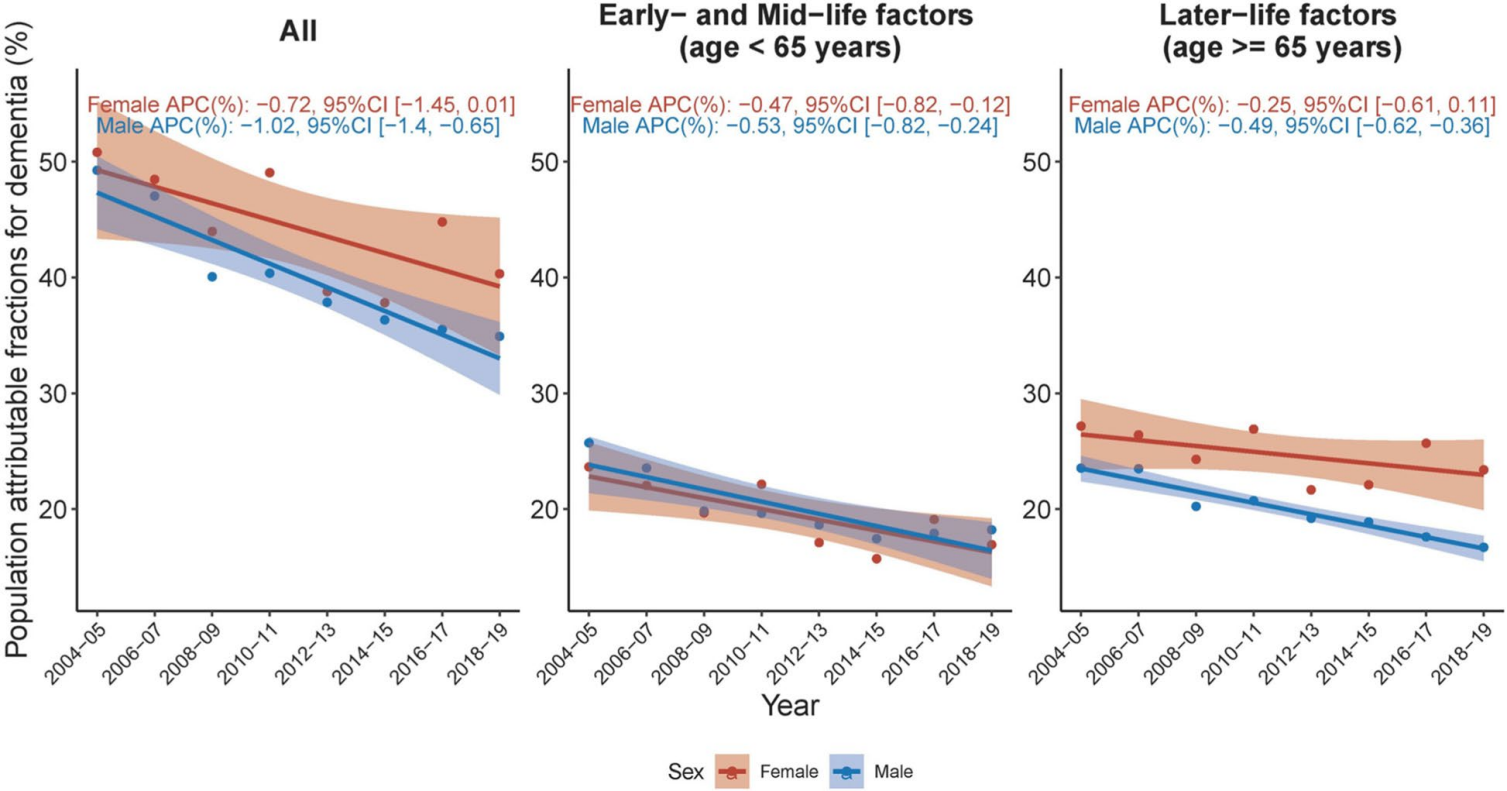
Temporal trends in population attributable fraction of 12 modifiable risk factors for dementia, 2004–2019, by sex. Average percentage change (APC) was used to quantify the temporal trend in population attributable fraction (PAF, as %), extracted from linear regression with PAF as the outcome and continuous form of year as the predictor. The APC indicates the extent to which the percentage points of PAF vary with each passing year

An analysis of overall PAF by sex revealed that women had a higher overall MRF PAF compared to men (Fig. 3, Panel C). This sex discrepancy was primarily due to the higher PAF of later-life factors among females (variance of PAF across sex = 90.4 in later-life factors vs 1.23 in early- and mid-life factors) (Fig. 3, Panel A and B). Despite both sexes showing a declining trend in overall MRF PAF from 2004 to 2019, the decrease was only statistically significant in males (APC -1.02, 95% CI [-1.40, -0.65]) but not in females (APC -0.72, 95% CI [-1.45, 0.01]) (Fig. 3, Panel C). The temporal pattern of the PAF associated with later-life factors mirrored the general trend (Fig. 3, Panel B). In the context of early- and mid-life factors, both females and males exhibited a significantly decreasing trend, with respective APC values of -0.47 (95%CI [-0.82, -0.12]) and -0.53 (95% CI [-0.82, -0.24]) (Fig. 3, Panel A). Differences in these temporal trends between sexes led to an expansion of the MRF PAF gap between females and males, which widened from 1.50 in 2004/2005 to 6.45 in 2018/2019.

**Fig. 4:**
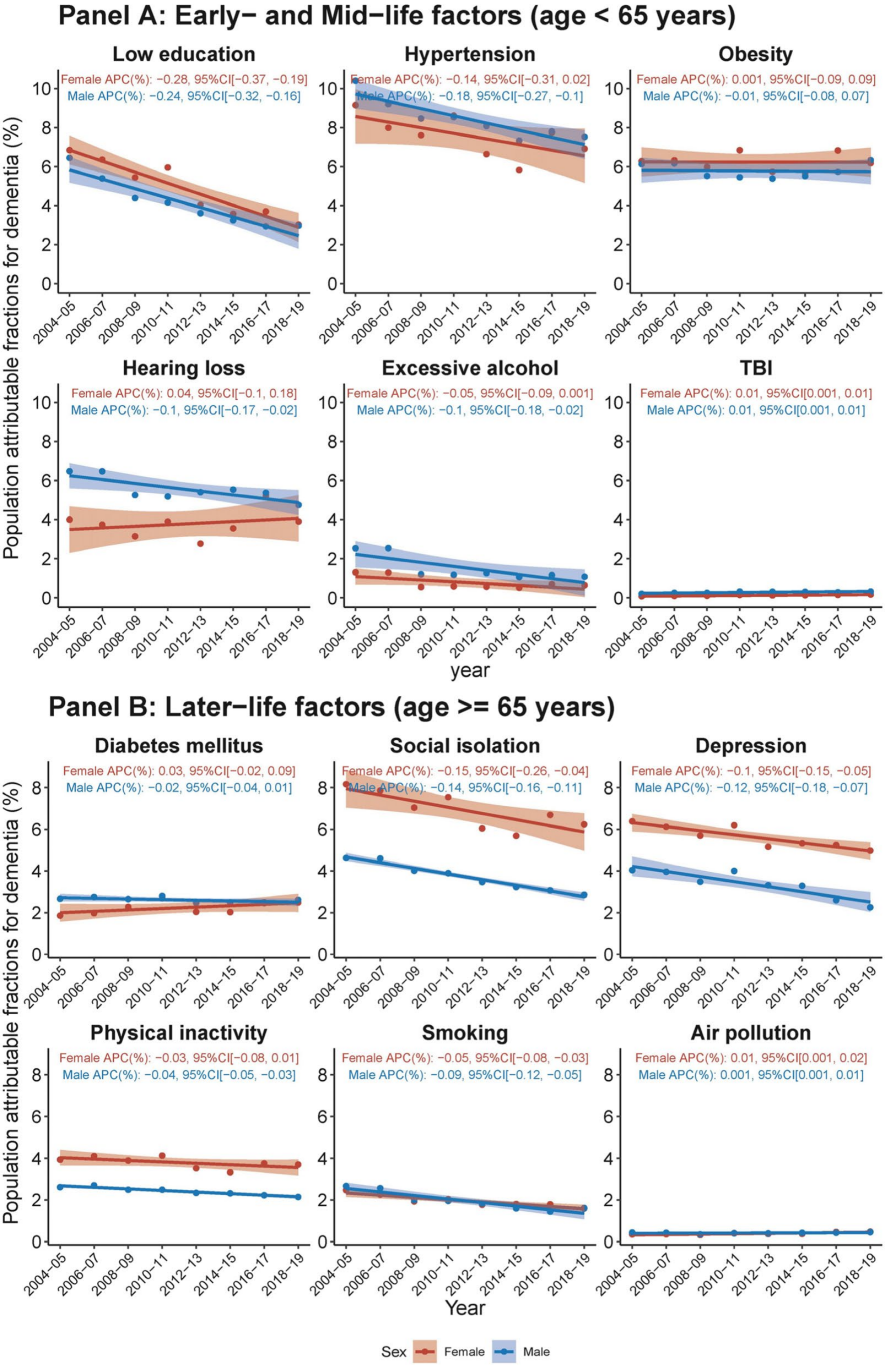
Temporal trends in population attributable fraction of 12 modifiable risk factors for dementia, 2004–2019, by sex and risk factor. Average percentage change (APC) was used to quantify the temporal trend in population attributable fraction (PAF, as %), extracted from linear regression with PAF as the outcome and continuous form of year as the predictor. The APC indicates the extent to which the percentage points of PAF vary with each passing year

Analysis of IW-PAFs by sex showed that females had higher average IW-PAFs for low education, obesity, social isolation, depression, and physical inactivity, while males had higher average IW-PAFs for hypertension, hearing loss, excessive alcohol, TBI, and diabetes mellitus (Fig. 4). Social isolation (variance of IW-PAF across sex = 41.8), depression (21.3), hearing loss (13.1), and physical inactivity (7.90) were the primary risk factors contributing to the sex gap. Over the 15-year period studied, the IW-PAFs for low education, social isolation, depression, and smoking demonstrated statistically significant downward trends in both males and females. Conversely, the IW-PAFs for TBI and air pollution exhibited upward trends in both sexes. The IW-PAFs for hypertension, hearing loss, excessive alcohol consumption, and physical inactivity revealed significant downward trends in males, but these trends were not statistically significant in females. The IW-PAFs associated with diabetes mellitus did not change significantly over time when each sex was considered separately (but increased overall, as described above).

### PAF trajectory by socioeconomic status

**Fig. 5:**
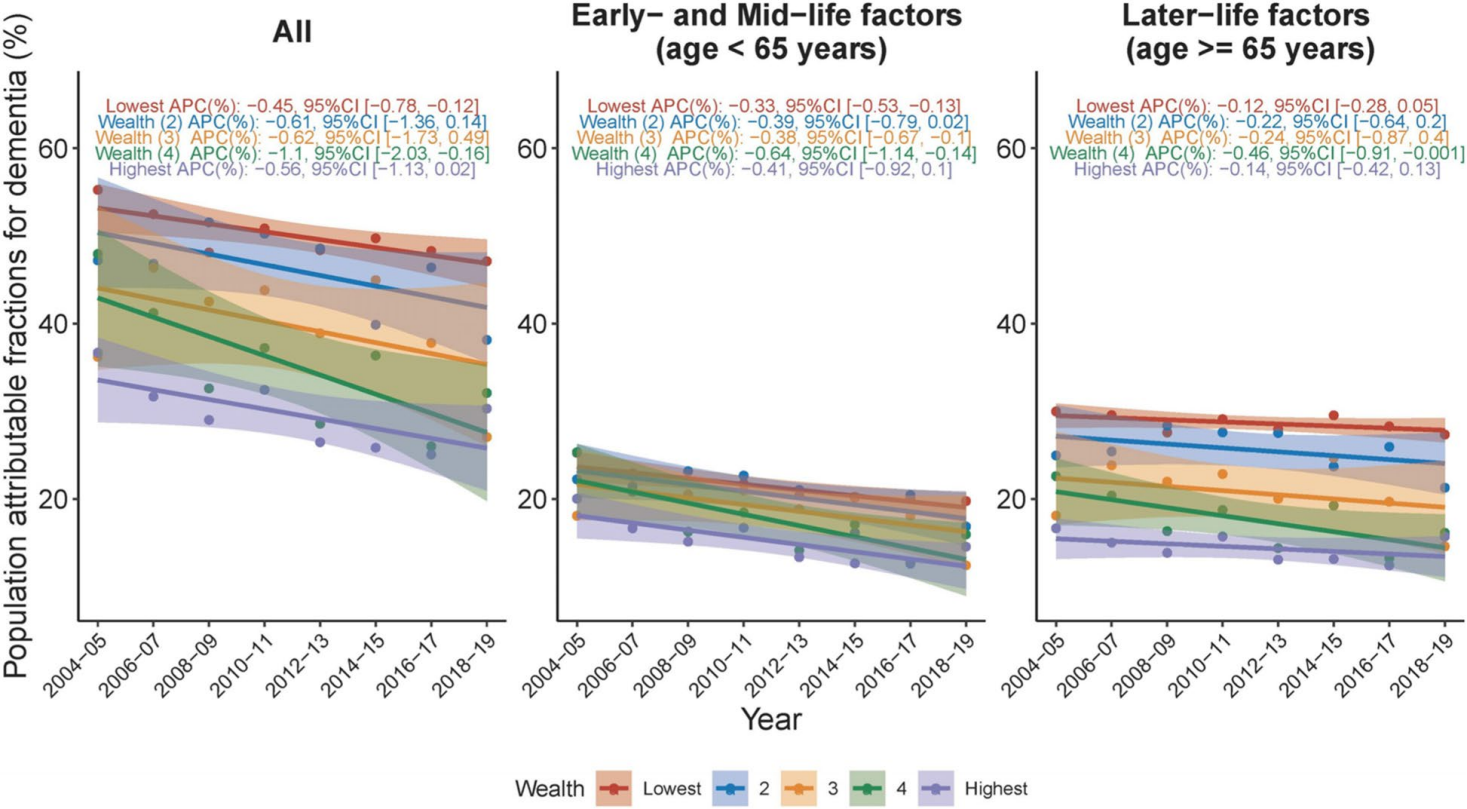
Temporal trends in population attributable fraction of 12 modifiable risk factors for dementia, 2004–2019, by socioeconomic status. Average percentage change (APC) was used to quantify the temporal trend in population attributable fraction (PAF, as %), extracted from linear regression with PAF as the outcome and continuous form of year as the predictor. The APC indicates the extent to which the percentage points of PAF vary with each passing year

An evaluation of overall MRF PAF by SES revealed a higher MRF PAF in lower-income groups compared to higher-income groups (Fig. 5, Panel C). This disparity across SES primarily stemmed from the variance of PAF associated with later-life factors (variance across SES groups = 283.41 in later-life factors vs 50.88 in early- and mid-life factors) (Fig. 5, Panel A and B). During 2004–2019, the decreasing trend in overall MRF PAF across SES groups was significant only in low and middle-high income groups (APC -0.45, 95% CI [-0.78, -0.12]; APC -1.10, 95% CI [-2.03, -0.16], respectively), thereby widening the gap between these two groups (7.21 in 2004/2005 and 15.0 in 2018/2019) (Fig. 5, Panel C).

**Fig. 6:**
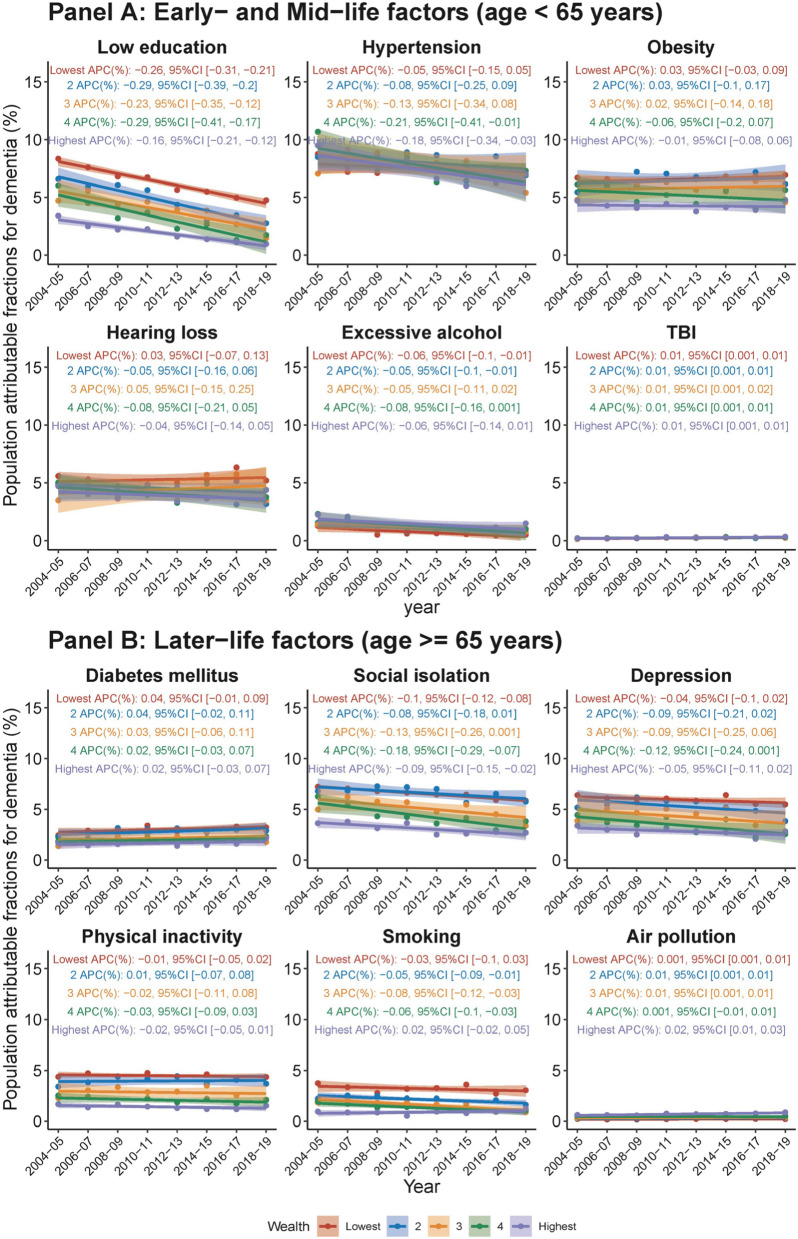
Temporal trends in population attributable fraction of 12 modifiable risk factors for dementia, 2004–2019, by socioeconomic status and risk factor. Average percentage change (APC) was used to quantify the temporal trend in population attributable fraction (PAF, as %), extracted from linear regression with PAF as the outcome and continuous form of year as the predictor. The APC indicates the extent to which the percentage points of PAF vary with each passing year

Upon examining the IW-PAF across SES and risk factors, most risk factors demonstrated a higher average IW-PAF in lower-income groups than in higher-income groups, except for excessive alcohol and air pollution, which were more dominant in higher-income groups (Fig. 6). Low education (variance of IW-PAF across SES groups = 23.20), social isolation (19.61), physical inactivity (13.75), depression (13.4), obesity (6.90) and smoking (6.56) were the primary factors that contributed to the disparity across SES groups (Fig. 6). Over the 15-year period studied, the temporal trend of each risk factor’s IW-PAF remained generally consistent across SES groups.

Sensitivity analyses, excluding cases with missing values instead of imputation them (Sup Figs. 1-6) and excluding those with probable dementia or those who responded by proxy during follow-up (Sup Figs. 7–12), all confirmed our primary results, in terms of temporal trends and the relative importance of each risk factor.

## Discussion

### Statement of principal findings

In England, although the PAF of modifiable risk factors for dementia showed a downward trend across 2004–2019, this temporal trend was slight and insignificant, with a substantial proportion of dementia cases (38%) still attributable, assuming causal relationships, to MRFs in 2018/2019. Hypertension has been the primary MRF driving the dementia burden, driving average 8.21% of cases of dementia during 2004–2019, followed by obesity (6.16%), social isolation (5.61%), hearing loss (4.81%), depression (4.72%), low education (4.63%), physical inactivity (3.26%), diabetes mellitus (2.49%), smoking (2.0%), excessive alcohol consumption (1.16%), air pollution (0.42%), and TBI (0.26%). However, only IW-PAFs of social isolation, low education, and smoking showed significant decreasing trends, while IW-PAFs of TBI, diabetes mellitus, and air pollution exhibited significant increasing trends. In contrast, the IW-PAFs of the remaining factors did not display any statistically significant changes over the study period. When disaggregated by sex, women have continuously higher PAF of MRFs than men, mainly attributed to later-life factors, notably social isolation, depression, and physical inactivity. Additionally, hearing loss, categorized as an early- to mid-life factor, also partially contributed to the observed sex disparity. Similar divergence was seen when examining PAF by SES, with people from low-income groups having continued higher PAF than those who are richer, also primarily linked to later-life factors, including social isolation, physical inactivity, depression, and smoking. Early- and mid-life factors, specifically low education and obesity, were also found to contribute moderately to the SES-based divergence in dementia risk. A critical examination of the temporal PAF or IW-PAF trends stratified by sex and SES suggest that the identified gaps across sex or SES have remained unchanged or increased over time.

### Interpretation

The finding that ~ 40% of dementia cases can be attributed to 12 MRFs as of 2018/2019 is in line with prior studies, such as the extensive study of the Lancet Commission [10]. Our study adds to previous work by describing the temporal trend of the PAFs, and suggesting that previous attempts to address MRFs have been at best only marginally effective and also sheds light on the direction of future preventative strategies. The persistence of MRFs in influencing incident cases of dementia may stem from various factors. First, some MRFs such as obesity are closely tied to behavioral environments, which are often entrenched and challenging to change at the population level [10]. Additionally, societal changes such as an ageing population and urbanization might indirectly exacerbate some risk factors such as air pollution or social isolation [43]. Nevertheless, the small downward trend observed in our study provides some optimism that changing risk factors is possible. This is supported by previous studies, which showed reductions in risk factors like blood pressure and smoking [27, 28]. It suggests that dementia prevention initiatives, such as those aimed at enhancing awareness and early detection of risk factors of dementia may have a positive but yet insufficient, unevenly distributed, or inadequately sustained effect. In addition, when our results are viewed in a global context, the UK's overall PAF for dementia MRFs (around 40%) falls in the middle-upper range of the spectrum—from Mozambique's 24% to Australia's 48% [6–9, 11–20]. This variation primarily reflect the disparate populations and the prevalence of risk factors across these regions. Nevertheless, the UK's middle-upper position further supports the possibility of improvement.

Our findings on IW-PAFs of 12 MRFs further provide crucial insights to guide prevention efforts. Notably, like other countries [8, 17], metabolic risk factors, like hypertension and obesity, were the top factors in England. Indeed, hypertension alone accounted for a striking average 8.67% of cases of dementia throughout our study period in England. These findings underscore the urgent necessity for healthcare professionals and public health policymakers to maintain, if not amplify, their focus on preventative strategies—from diet and lifestyle modifications to appropriate antihypertensive therapies [44, 45]. Evidence from earlier work, which registered a decrease in dementia incidence over the past three decades partly due to efficient cardiovascular risk management [46], lends weight to the potential efficacy of this approach. Its cost-effectiveness was also validated by a UK-based modelling study [44] and addressing risk factors such as hypertension would have positive health benefits beyond dementia, for example in decreasing the incidence of heart disease or stroke.

However, it is also important to consider the potential impact of decreasing mortality in old age as a result of advancements in the prevention of dementia, particularly concerning hypertension. A recent simulation modelling study by Chen et al. (2023) explored the effects of changes in future hypertension prevalence on mortality, dementia, and disability simultaneously in England and Wales [47]. The study found that if the downward hypertension prevalence trend accelerates, with prevalence falling by 50% between 2017 and 2060, there would be a modest reduction in deaths and a small increase in dementia burden. This suggests that the beneficial effect of lower population blood pressure distribution on the incidence of dementia might not offset the expansion of the susceptible population due to reduced mortality. Therefore, while targeting hypertension remains crucial for dementia prevention, policymakers should also consider the potential trade-offs and plan accordingly to ensure adequate resources and support for an ageing population with potentially increased dementia prevalence.

The decrease in IW-PAFs of social isolation, low education, and smoking likely reflects the successes of public health interventions or national policy (for example in education) in these areas, yet their persistent contribution to the dementia burden emphasizes the need for sustained efforts. However, the upward trend or stagnation of other IW-PAFs is worrisome and diverges from the desire to decrease dementia risk via better management of modifiable factors [10, 24], especially given that cost-effective interventions have been identified for some factors, such as hearing aids for hearing loss [48]. This may suggest possible challenges, such as late detection, inadequate management of these conditions, or broader societal and environmental changes impacting these risk factors. Studies specifically examining each risk factor are needed.

Our findings on IW-PAFs of 12 MRFs, including their temporal trends and corresponding differences between early- and mid-life factors and later-life factors, underscore the importance of collective-level interventions using a life course approach. Such interventions that reduce population-level exposure to risks across the lifespan could more effectively mitigate an individual’s likelihood of developing dementia and related conditions, compared to interventions targeting sole risk factors [49]. For instance, age-related hearing loss demonstrates cumulative risk patterns beginning in early life. Similarly, hypertension risk correlates with behaviors like excessive salt intake and physical inactivity starting in youth that tracks into older age. In addition to the pharmaceutical treatments aforementioned, implementing upstream social and structural interventions at the population level that address modifiable dementia risk factors would likely have more impact than downstream individual-level interventions focused narrowly on diet and lifestyle changes alone [50].

The disaggregation of our findings by sex highlighted that there is more potential to prevent dementia cases in women than men at the moment. Similar sex disparity was also identified in Chile [17]. In our study, later-life factors were the primary drivers of sex disparity. Specifically, females demonstrated a higher overall MRF PAF, predominantly driven by social isolation, depression, and physical inactivity – later-life factors that are often intricately intertwined with gender roles, societal expectations, and ageing, but which are all modifiable. Females, particularly in their later years, maybe more susceptible to social isolation due to factors such as widowhood or caring for family members, which aligns with the findings of a recent study by Santini et al. (2020) that pointed to a higher prevalence of loneliness in older women [51]. Additionally, a systematic review by Guthold et al. (2018) showed a greater risk of physical inactivity among women [52], which is concerning considering our results. Similarly, a contribution from hearing loss, an early- to mid-life factor, to the gender disparity observed, supports the findings of the Lancet Commission [10], which highlighted the impact of hearing loss on increased dementia risk among women. Therefore, efforts to improve social support networks and mental health services, to encourage physical activity, and to identify hearing loss early and treat it, may need to be tailored differently for men and women, emphasizing more on women in these campaigns.

Our analyses by the SES quintile revealed that the burden of modifiable dementia risk is shouldered disproportionately by low-income groups, suggesting a higher potential for preventative measures within this demographic. This socio-economic divergence in dementia risk can also be attributed predominantly to later-life factors such as social isolation, physical inactivity, depression, and smoking. Early- and mid-life factors like low education and obesity also contributed, albeit moderately, to this SES disparity. This finding aligns with existing literature indicating a strong link between socio-economic status and health outcomes, including dementia risk [53]. What sets our study apart is that we present a detailed picture of the individual contributions of each risk factor to SES disparity, thereby informing priorities for intervention. Specifically, collective-level interventions that address education, social isolation, depression, and lifestyle-related factors like obesity, physical inactivity, and smoking, may require greater focus on lower-income groups.

Our study further extends the findings on sex and SES disparities by providing a temporal perspective. Crucially, the persistence or even widening of these disparities across sex and SES over time, as indicated by our temporal PAF or IW-PAF trends, warrants urgent attention as suggested above. The disparities we observed are consistent with those reported in studies on disability-free life expectancy in the UK [54, 55]. While the disparity itself is not a new discovery, our study uniquely contributes evidence specific to dementia. This stagnation or widening of the gaps contrasts with the fundamental principles of public health interventions, which envision an equitable and fair healthcare system. Notably, our findings revealed a diverging pattern between the lowest and 4th wealth quintiles, with a notable decline observed for the 4th quintile but not for the highest quintile. Our further by risk analysis indicted that compared to the highest quintile, the 4th quintile had a relatively higher decline in IW-PAF for low education, obesity, hearing loss, social isolation, depression, and smoking. This finding suggests that individuals in the 4th quintile may have benefited more from public health interventions targeting these risk factors than those in the highest quintile. It is possible that the 4th quintile had a higher initial prevalence of these risk factors, allowing for a greater margin of improvement. Additionally, public health interventions may have been more effective in reaching and influencing individuals in the 4th quintile, possibly due to factors such as health literacy and access to community-based programs, which can vary across socioeconomic groups [56]. We suggest future research examines the effectiveness of existing interventions and seeks innovative solutions to address these persistent disparities, with a particular focus on the gaps identified by our IW-PAF metrics. Whilst intervention for some risk factors may be difficult or may be delayed in impact for others (e.g. education), for some (e.g. hypertension) there are established mechanisms for identification and treatment that should be achievable within current healthcare settings. Our work presented here underscores the need for determined, consistent, up-to-date and targeted strategies to tackle these risk factors. Our work also stresses the importance of enhancing prevention efforts directed at women and individuals in low-income groups. These will be an essential part of ameliorating the dramatically increasing personal, economic and social costs arising from dementia.

### Strengths and limitations

In addition to being the most up-to-date examination of this issue in the UK, our study brings sub-group and longitudinal views on modifiable risk factors for dementia, delivering several significant advances. Firstly, this study, to the best of our knowledge, is the first to map out the temporal trend of the MRF PAF for dementia in England from 2004–2019, hence providing a nuanced understanding of the evolution of dementia risk factors over a 15-year period. This longitudinal perspective not only offers a comprehensive view of the changing patterns of dementia risk factors but also delivers an evaluation of the effectiveness of past public health interventions. Secondly, our research extends the analysis by examining the role of sex and SES in dementia risk. We examine not just the overall disparity but also the differential contributions of each risk factor to this disparity. This sex- and SES-specific analysis allows us to explore underlying socioeconomic and gender issues tied to dementia risk, providing a more comprehensive understanding of the inequities in dementia incidence. Thirdly, the data and methodologies employed are robust. The RRs for each risk factor, sourced from recent meta-analyses, gather up the most compelling evidence currently available [10, 24]. Although these RRs do not adjust mutually for all other risk factors, we have ensured accounting for the non-independence of these factors via communality weights, a method known for its conservative accuracy in estimating the combined PAF. And unlike other studies, we have sourced all analysis components, including prevalence, communalities, and overall PAF, from a single information source, thereby maintaining high internal consistency, a factor not usually seen in other studies.

Several limitations need to be considered when interpreting our findings. First, the use of PAF posits a theoretical scenario where dementia risk can be wholly eliminated by removing risk factors. While entirely eradicating these factors is unfeasible, any reduction should theoretically forestall or prevent the onset of dementia, thereby decreasing prevalence. However, the PAF model does not account for potential increases in dementia prevalence due to longevity resulting from risk factor reduction [8]. Thus, our PAF-derived estimates of prevalence reduction might be overestimated. Nevertheless, it is also possible that those who live longer due to mitigating the twelve risk factors are less likely to develop dementia, so the age-related prevalence is decreased. A trend of diminishing dementia incidence has been observed in numerous countries over recent decades [21, 22], notwithstanding the potential augmentation in risk due to the simultaneous ageing of the population within this timeframe. In addition, one universal limitation in PAF studies pertains to the dichotomous presentation of relative risk data, as opposed to a continuous association between the magnitude of the risk factor and dementia risk [23]. Another universal limitation in PAF studies is the lack of consideration of the time lags between the measurement of risks and actual outcome in analysis. Moreover, our study could not distinguish the extent to which changes in PAF stem from shifting prevalence versus alterations in the shared variance among risk factors, given the assumption of constant relative risks over time. Addressing these three limitations will require the development of advanced statistical methods in future efforts.

Second, our study also faced limitations due to the lack of precise data on the prevalence of factors of air pollution, social isolation, TBI, and hearing loss. Direct measures of air pollution exposure and social isolation were unavailable, prompting us to employ proxies. The use of household fuel types as a proxy for air pollution, and cohabitation status for social isolation, has inherent drawbacks. For example, using home fuel types only addresses indoor air pollution, neglecting the outdoor aspect, and using cohabitation as a proxy for social isolation assumes that those who live alone have less social contact, although the increased risk of dementia in lifelong singles compared to married people [57] suggests this is reasonable. Another concern is that the ELSA did not include data on TBI, necessitating us to rely on average communality measures from other variables, as per the standard practice in dementia PAF research [10]. Moreover, TBI prevalence was estimated from the incidence rate of hospital admissions for head injuries with ICD10 codes S02, S04, S06, S07, S09, T04.0, and T06.0, which might underestimate its true prevalence, and thus potentially distort its PAF. Fortuitously, paralleling findings from other studies [6, 18], the contribution of TBI to the dementia population risk appears to be minimal; thus, any potential bias introduced due to our method of estimating TBI prevalence is unlikely to affect the practical implications of our results substantially. Moreover, the self-reported or observation-based measure of hearing loss used in our study may not align with the more stringent 25 dB threshold criterion [24], potentially leading to an underestimation of its contribution.

Third, the dataset we used is constrained by the presence of missing data. However, the robustness of our primary findings was affirmed by sensitivity analyses.

Fourth, the indicators used in our study do not encapsulate all potential hazards, such as accessibility of healthcare services [58]. The 12 MRFs used, pinpointed by the Lancet Commission for the general population, may not entirely elucidate the heightened dementia risk within socioeconomically disadvantaged demographics, as suggested by prior studies [9, 59].

Fifth, reverse causation could represent a potential confounding factor. For instance, depression could either precede or result from dementia, rather than being a causative agent [12, 14]. However, our sensitivity analyses, excluding participants with possible dementia during follow-up, supported our primary analyses.

Sixth, participants had the opportunity to engage in multiple iterations of the ELSA, it is important to note that, despite ELSA providing sampling weights to ensure representativeness in each wave, the representational efficacy of this approach may not be as robust as that achieved through a newly conducted cross-sectional survey. As indicated in Sup Table 2, it appears that the sampling weights over-adjusted the population, resulting in a younger adjusted demographic. This could potentially lead to an underestimation of the PAF. Nevertheless, this utilization is acceptable given that a recent study successfully projected the number of people with dementia in England up to 2040 based on ELSA data [33]. However, it is crucial to acknowledge the potential for survival bias in our study, which may lead to an underestimation of the PAF, particularly among individuals with lower SES. Considering the typically higher mortality rates observed among those with lower SES [55], as well as the fact that some of the MRFs we focused on (such as physical inactivity and smoking) were more concentrated in this subgroup [60], there is a possibility that participants with more risk factors were more likely to die during the study period. This could result in an underestimation of the corresponding prevalence and PAF disparities.

Seventh, the identification of probable dementia was based on a 25-point cognitive scale and a threshold of 1.5 standard deviations (SDs). Although this scale and cut-off have been extensively employed in studies within the United Kingdom [33, 61, 62], their validation is primarily documented in the United States [63, 64]. Notwithstanding this geographic specificity in validation, it is noteworthy that both our principal analysis and sensitivity analysis converged on the same conclusion.

Finally, our study was based on data collected prior to the COVID-19 pandemic, a condition with increased mortality and poor outcomes in older adults and those with cognitive impairment. How this might impact risk factors and incidence of dementia is unknown and not covered in the data reported here.

## Conclusions

Through a comprehensive and longitudinal analysis spanning 15 years, we have provided fresh insights into the evolution of modifiable risk factors for dementia and their interplay with variables such as sex and SES. Our data and methodologies, anchored in the most recent meta-analyses, ensure a high degree of internal consistency. However, our results also illuminate potential directions for future research, including refining measurements for social isolation, assessing additional risk factors, looking at post-pandemic populations, and addressing data limitations. Despite these considerations, our findings have meaningful implications for practice to ensure that future prevention strategies cater to the unique needs and challenges of different groups.

### Supplementary Information

Supplementary Material 1.

## Data Availability

The data are publicly available and can be accessed here (https://www.elsa-project.ac.uk/).

## Abbreviations

MRFs: Modifiable risk factors
CAPI: Computer-Assisted Personal Interviews
DALY: Disability-adjusted life years
ELSA: English Longitudinal Study of Ageing
HSE: Health Survey for England
IW-PAFs: Individual weighted PAFs
OHID: Office for Health Improvement and Disparities
PAFs: Population attributable fractions
RR: Relative risk
SES: Sex and socioeconomic status
TBI: Traumatic brain injury
UK: United Kingdom
