# Supplementary material for: Temporal trends in population attributable fractions of modifiable risk factors for dementia: a time-series study of the English Longitudinal Study of Ageing (2004–2019)
Source: BMC Med. 2024 Jun 26;22:268. doi: 10.1186/s12916-024-03464-2 (PMC11210022; doi:10.1186/s12916-024-03464-2)

**Supplementary: The transformation between prevalence and incidence rate**

The U.S. Centers for Disease Control and Prevention suggested that when the frequency of disease is rare (specifically, less than 10% of the population being affected), a correlation exists between the prevalence and incidence rate, which can be mathematically represented as:

$$Prevalence = Incidence rate \times Average duration of disease$$

Within the context of this study, the average duration of the disease was determined through the formula:

$$Average duration of disease = Average life expectancy in the England - Average years of life lost due to traumatic brain injury$$

Where, the parameter 'Average Years of Life Lost due to Traumatic Brain Injury' was derived from a study conducted by Marek Majdan, published in PLOS Medicine (DOI: 10.1371/journal.pmed.1002331); the 'Average Life Expectancy in England', categorized by age, sex, and year, was sourced from the “National Life Tables – Life Expectancy in the UK”, as published by the UK Office for National Statistics (<https://www.ons.gov.uk/peoplepopulationandcommunity/birthsdeathsandmarriages/lifeexpectancies/bulletins/nationallifetablesunitedkingdom/2018to2020#:~:text=Across%20the%20UK%2C%20life%20expectancy,years%20for%20females%20in%20Northern>).

**Sup Table 1. Relative risks between factors and dementia incidence**, extracted from the report published by the Lancet Commission in 2020 (DOI: 10.1016/S0140-6736(20)30367-6).

|  | Risk ratio (95% confidence interval) |
| --- | --- |
| **Risk factors** |  |
| **Early-life (age <45 years)** |  |
| Low education (<= 65) | 1.59 (1.26-2.01) |
| **Midlife (age 45–64 years)** |  |
| Hearing loss | 1.94 (1.38-2.73) |
| Hypertension | 1.60 (1.16-2.24) |
| Obesity | 1.60 (1.34-1.92) |
| Excessive alcohol | 1.18 (1.06-1.31) |
| Traumatic brain injury (TBI) | 1.84 (1.54-2.20) |
| **Later-life (age ≥65 years)** |  |
| Smoking | 1.60 (1.15-2.20) |
| Depression | 1.90 (1.55-2.33) |
| Physical inactivity | 1.38 (1.16-1.67) |
| Social isolation | 1.57 (1.32-1.85) |
| Diabetes mellitus | 1.50 (1.33-1.79) |
| Air pollution | 1.09 (1.07-1.11) |

**Sup Table 2**. **Weighted description of socio-demographic factors and modifiable risk factors by survey year.** The data are presented as percentages, and the p-values were obtained from weighted logistic regression analyses, with the variable in each row serving as the outcome and the year as the predictor.

| **Variable** | **Year (= 2004-05) (n = 9365)** | **Year (= 2006-06) (n = 9671)** | **Year (= 2008-09) (n = 10923)** | **Year (= 2010-11) (n = 10122)** | **Year (= 2012-13) (n = 10437)** | **Year (= 2014-15) (n = 9500)** | **Year (= 2016-17) (n = 8308)** | **Year (= 2018-19) (n = 8578)** | P |
| --- | --- | --- | --- | --- | --- | --- | --- | --- | --- |
| **Socio-demographic factors** |  |  |  |  |  |  |  |  |  |
| Age (>=65) | 52.70% | 47.80% | 45.90% | 52.40% | 43.80% | 44.20% | 48.60% | 45.80% | < 0.001 |
| Sex (= Female) | 54.10% | 53.30% | 53% | 53.10% | 52.70% | 52.50% | 52.70% | 52.50% | 0.105 |
| Wealth status |  |  |  |  |  |  |  |  |  |
| Lowest | 21.40% | 21% | 22% | 21.80% | 23.60% | 23.60% | 23.70% | 24% | < 0.001 |
| 2 | 20.40% | 20.2% | 21% | 20.7% | 20.2% | 21% | 20.4% | 21.20% | 0.453 |
| 3 | 19.60% | 20.2% | 19.9% | 20% | 19.3% | 19.2% | 19.5 | 19.10% | 0.157 |
| 4 | 19.70% | 19.9% | 19.2% | 19.3% | 18.6% | 18.6% | 18.70% | 18.30% | 0.009 |
| Highest | 18.90% | 18.7% | 18% | 18.2% | 18.2% | 17.7% | 17.7% | 17.4% | 0.014 |
| **Modifiable risk factors** |  |  |  |  |  |  |  |  |  |
| Low education (= yes) | 46.90% | 42.8% | 40.6% | 38.7% | 32.1% | 29.6% | 27.9% | 24.4% | < 0.001 |
| Hypertension (= yes) | 64.10% | 59.2% | 61.2% | 62% | 57.1% | 54% | 56.7% | 54.1% | < 0.001 |
| Obesity (= yes) | 29.50% | 30.6% | 32.2% | 32.2% | 31.9% | 32.6% | 32.3% | 33% | < 0.001 |
| Hearing loss (= yes) | 24.60% | 22.9% | 21.3% | 22.2% | 21% | 24% | 23.9% | 21.9% | 0.422 |
| Excessive alcohol (= yes) | 20.80% | 22.1% | 12.9% | 12.5% | 13.5% | 12.2% | 13.1% | 12.9% | < 0.001 |
| Diabetes mellitus (= yes) | 9.60% | 9.8% | 12% | 13% | 10.7% | 11.3% | 12.2% | 12.7% | < 0.001 |
| Social isolation (= yes) | 26% | 24.6% | 24.6% | 24.7% | 19.8% | 18.9% | 19.6% | 19% | < 0.001 |
| Depression (= yes) | 16.70% | 18.1% | 18.8% | 19.4% | 19% | 18.4% | 14% | 14.6% | < 0.001 |
| Physical inactivity (= yes) | 17.90% | 18% | 19.2% | 19.4% | 17.8% | 17.2% | 16.8% | 16.9% | 0.002 |
| Smoking (= yes) | 15.80% | 15.6% | 15% | 13.9% | 14.1% | 13.1% | 11.1% | 11.6% | < 0.001 |
| Air pollution (= yes) | 12.80% | 12.9% | 13.4% | 14.1% | 15.4% | 16.5% | 16.3% | 17.1% | < 0.001 |

**Sup Figure 1. Overall temporal trends in population attributable fraction of 12 modifiable risk factors for dementia, no imputation**. Average percentage change (APC) was used to quantify the temporal trend in population attributable fraction (PAF, as %), extracted from linear regression with PAF as the outcome and continuous form of year as the predictor. The APC indicates the extent to which the percentage points of PAF vary with each passing year.


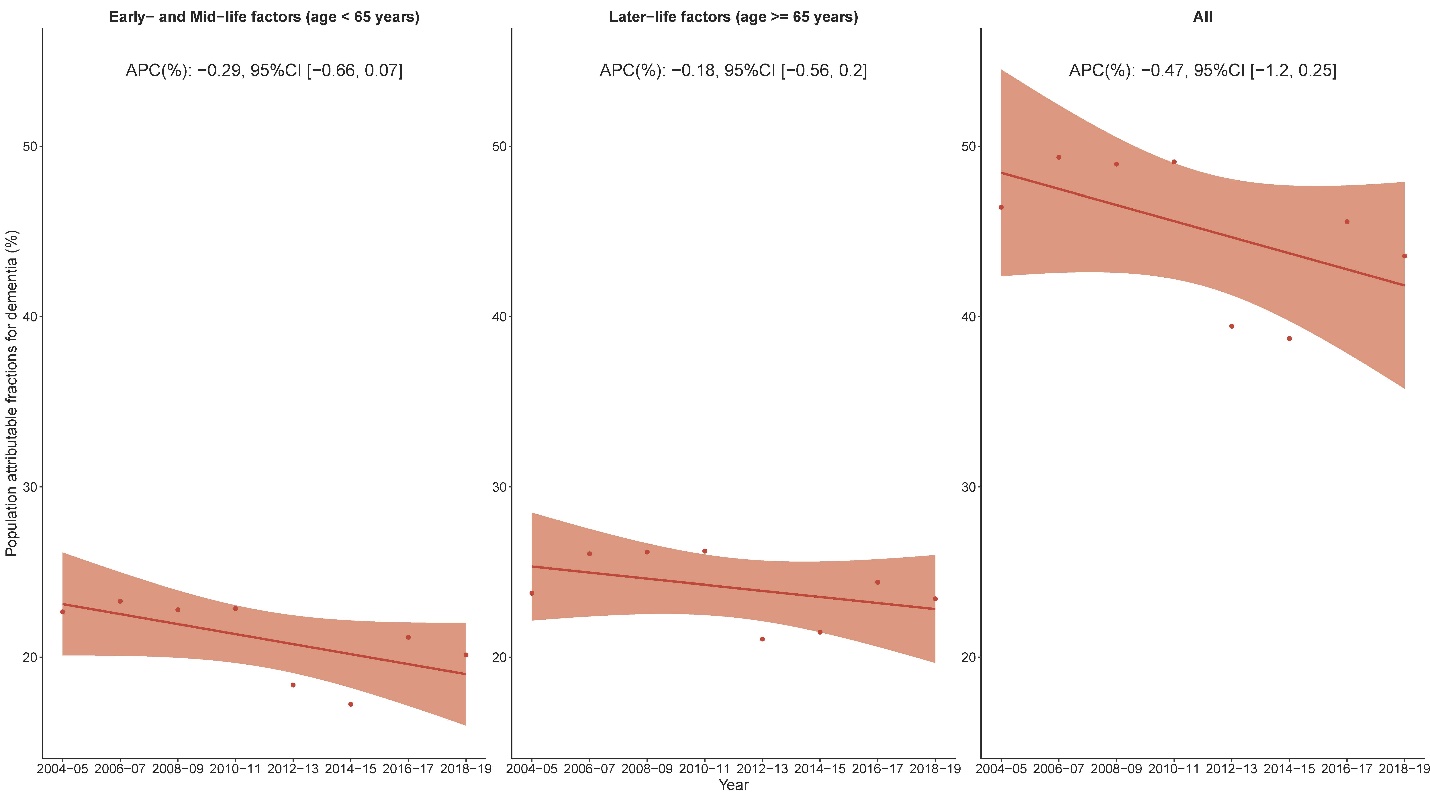


**Sup Figure 2. Temporal trends in population attributable fraction of 12 modifiable risk factors for dementia, by risk factor, no imputation.** Average percentage change (APC) was used to quantify the temporal trend in population attributable fraction (PAF, as %), extracted from linear regression with PAF as the outcome and continuous form of year as the predictor. The APC indicates the extent to which the percentage points of PAF vary with each passing year.


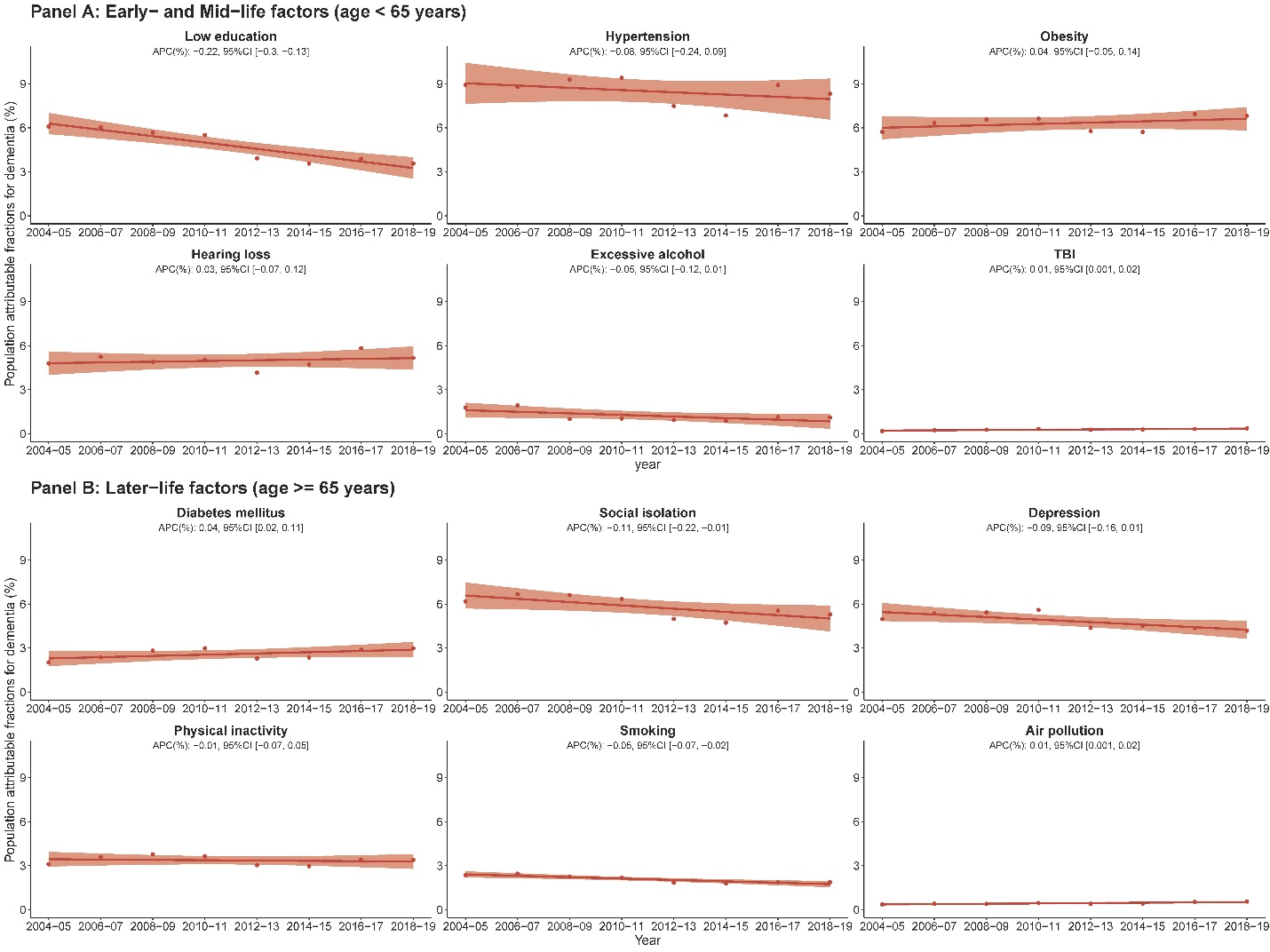


**Sup Figure 3. Temporal trends in population attributable fraction of 12 modifiable risk factors for dementia, by sex, no imputation.** Average percentage change (APC) was used to quantify the temporal trend in population attributable fraction (PAF, as %), extracted from linear regression with PAF as the outcome and continuous form of year as the predictor. The APC indicates the extent to which the percentage points of PAF vary with each passing year.


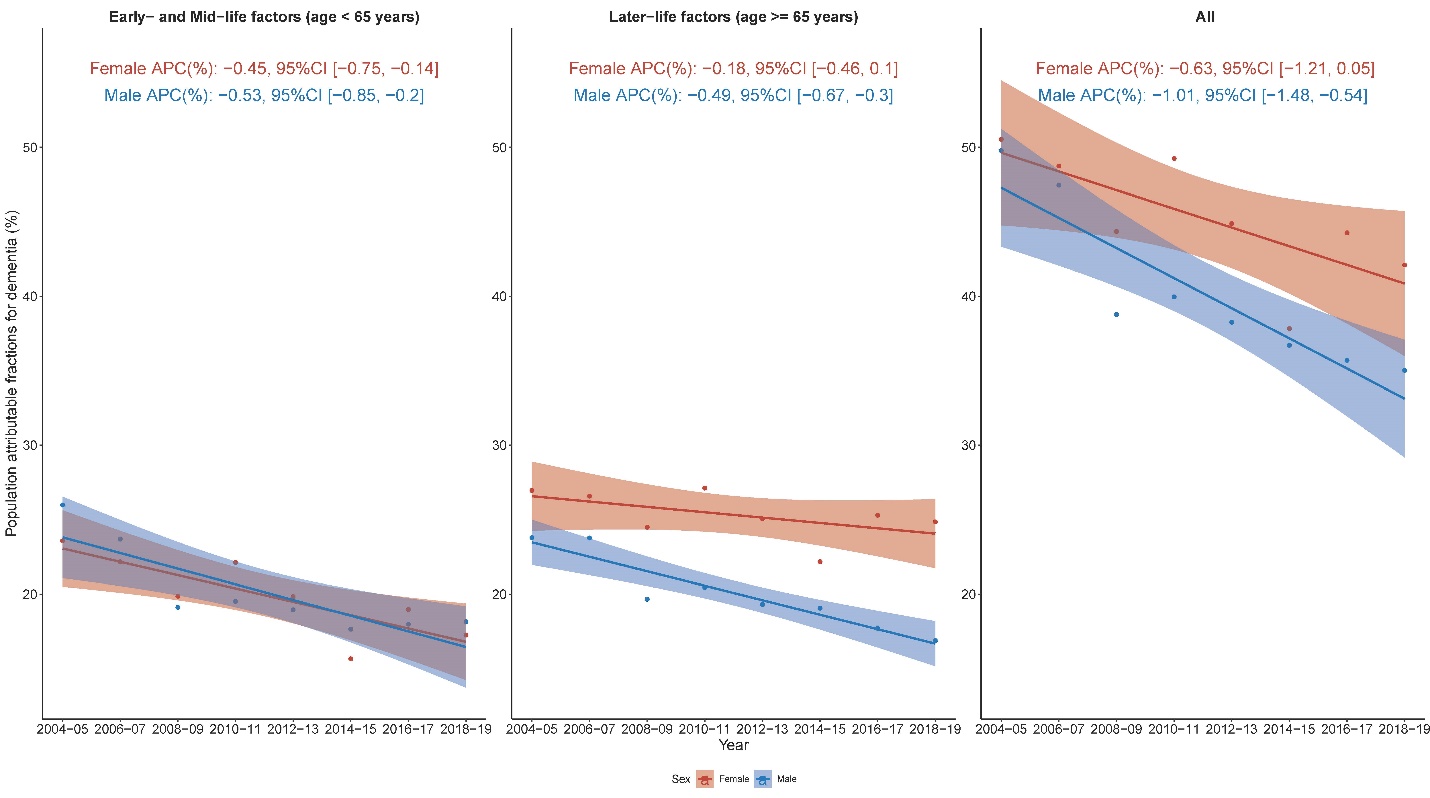


**Sup Figure 4. Temporal trends in population attributable fraction of 12 modifiable risk factors for dementia, by sex and risk factor, no imputation.** Average percentage change (APC) was used to quantify the temporal trend in population attributable fraction (PAF, as %), extracted from linear regression with PAF as the outcome and continuous form of year as the predictor. The APC indicates the extent to which the percentage points of PAF vary with each passing year.


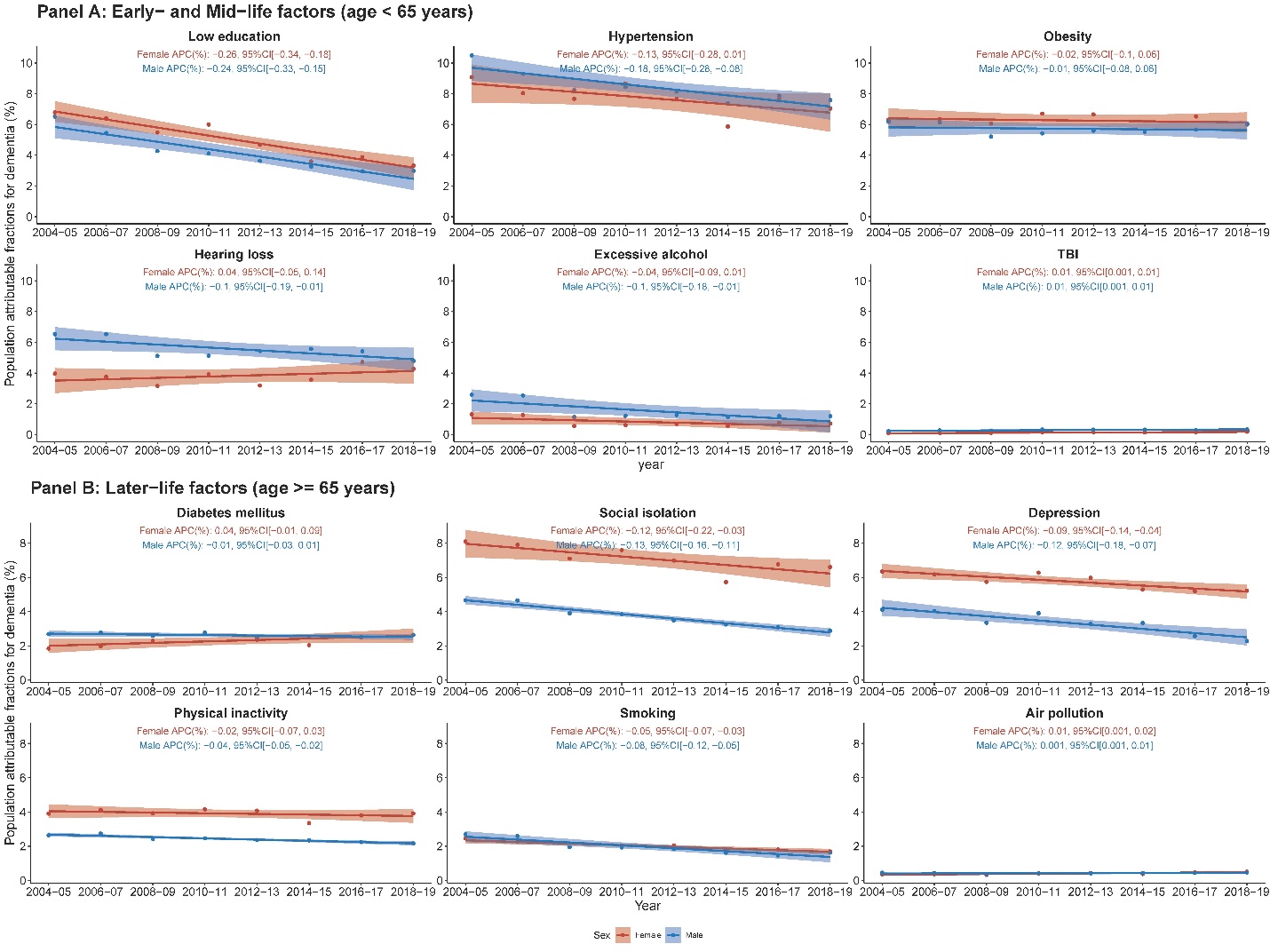


**Sup Figure 5. Temporal trends in population attributable fraction of 12 modifiable risk factors for dementia, by socioeconomic status, no imputation.** Average percentage change (APC) was used to quantify the temporal trend in population attributable fraction (PAF, as %), extracted from linear regression with PAF as the outcome and continuous form of year as the predictor. The APC indicates the extent to which the percentage points of PAF vary with each passing year.


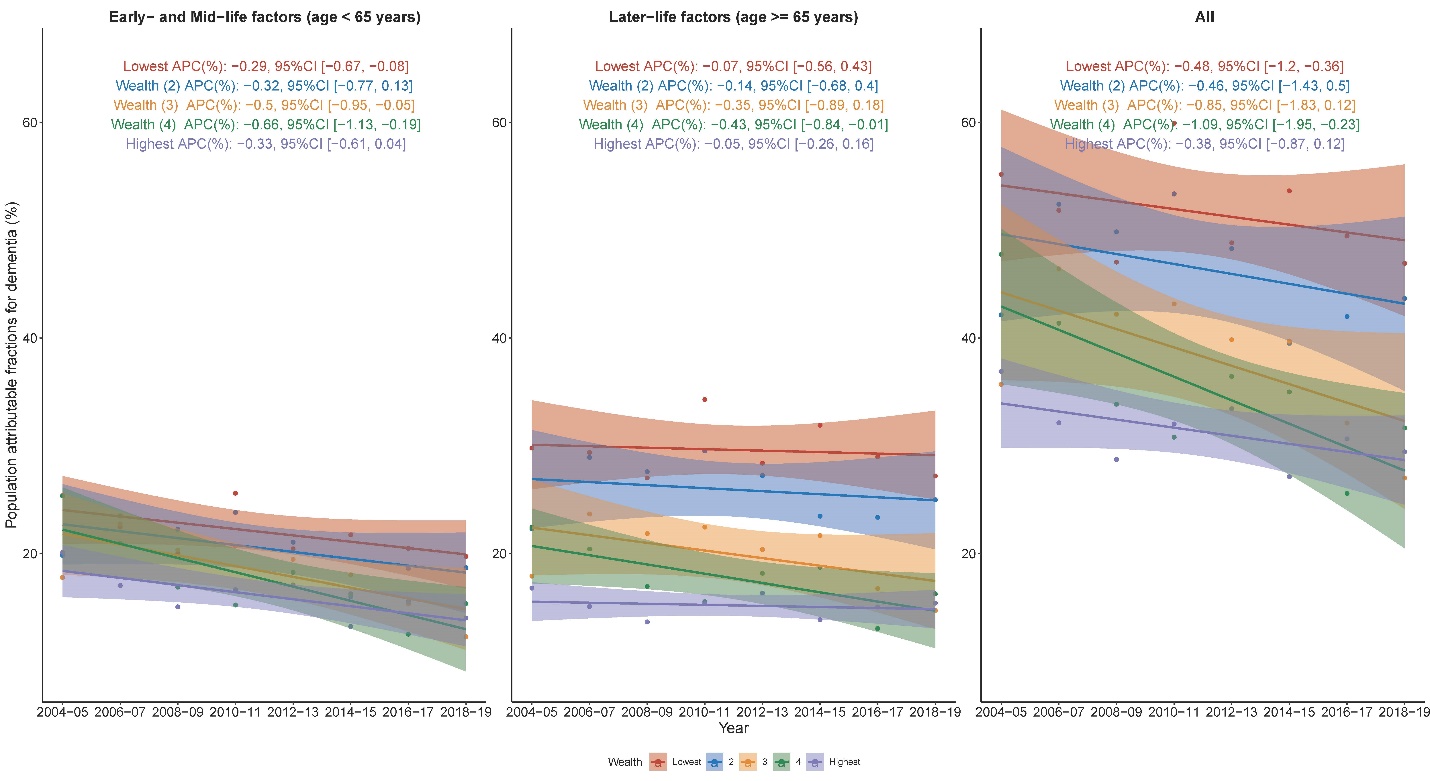


**Sup Figure 6. Temporal trends in population attributable fraction of 12 modifiable risk factors for dementia, by socioeconomic status and risk factor, no imputation.** Average percentage change (APC) was used to quantify the temporal trend in population attributable fraction (PAF, as %), extracted from linear regression with PAF as the outcome and continuous form of year as the predictor. The APC indicates the extent to which the percentage points of PAF vary with each passing year.


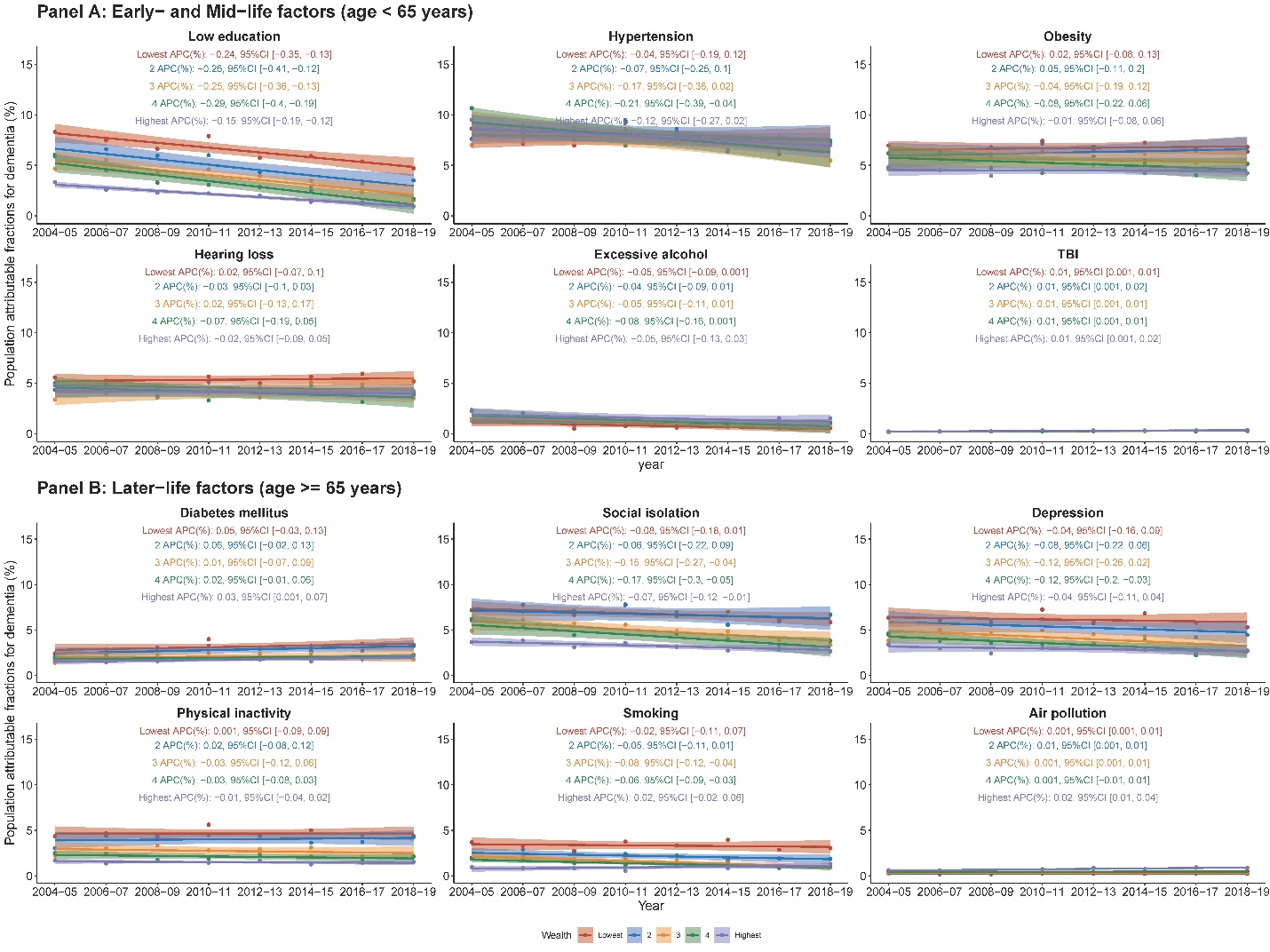


**Sup Figure 7. Overall temporal trends in population attributable fraction of 12 modifiable risk factors for dementia, excluding those with probable dementia or those responded by proxy during follow-up.** Average percentage change (APC) was used to quantify the temporal trend in population attributable fraction (PAF, as %), extracted from linear regression with PAF as the outcome and continuous form of year as the predictor. The APC indicates the extent to which the percentage points of PAF vary with each passing year.


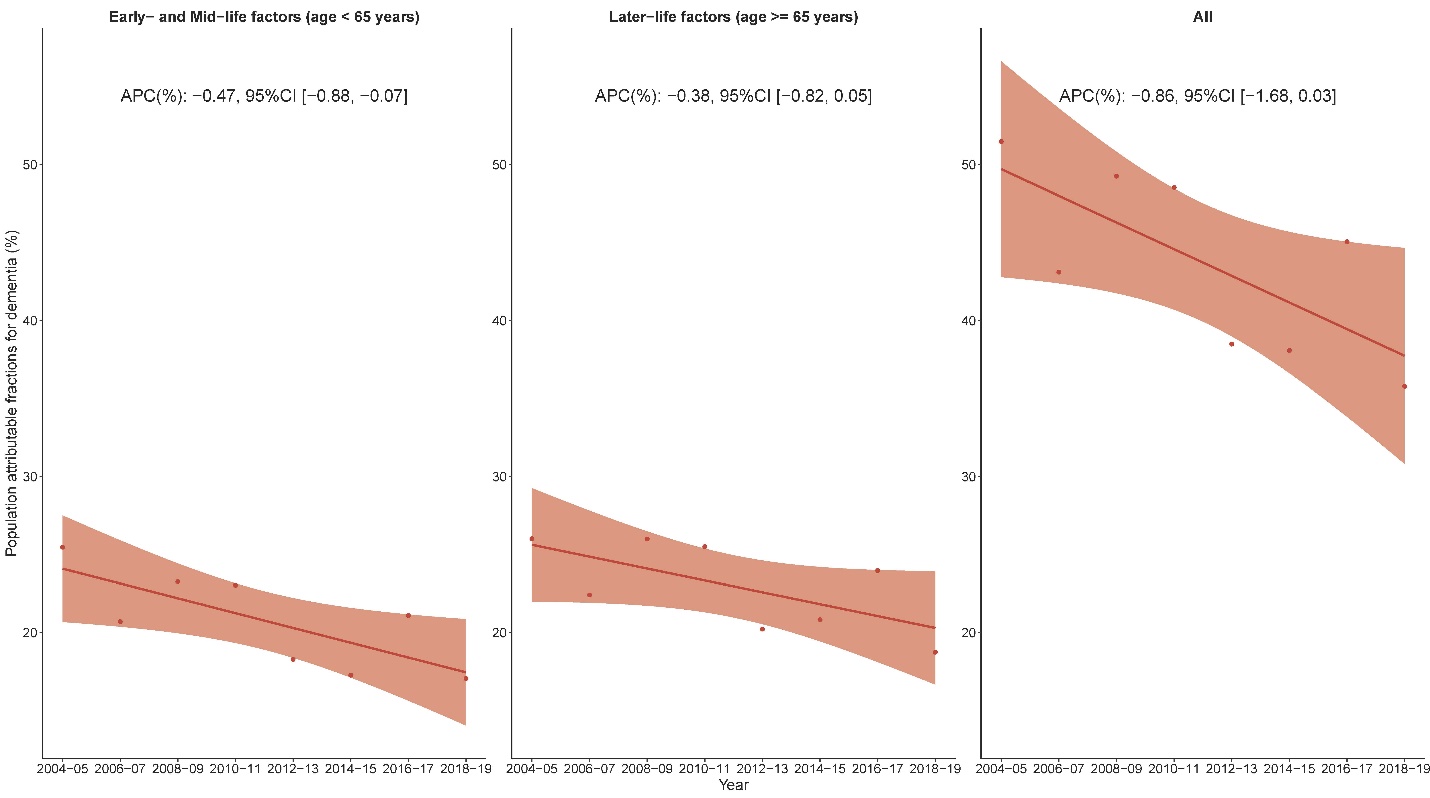


**Sup Figure 8. Temporal trends in population attributable fraction of 12 modifiable risk factors for dementia, by risk factor, excluding those with probable dementia or those responded by proxy during follow-up.** Average percentage change (APC) was used to quantify the temporal trend in population attributable fraction (PAF, as %), extracted from linear regression with PAF as the outcome and continuous form of year as the predictor. The APC indicates the extent to which the percentage points of PAF vary with each passing year.


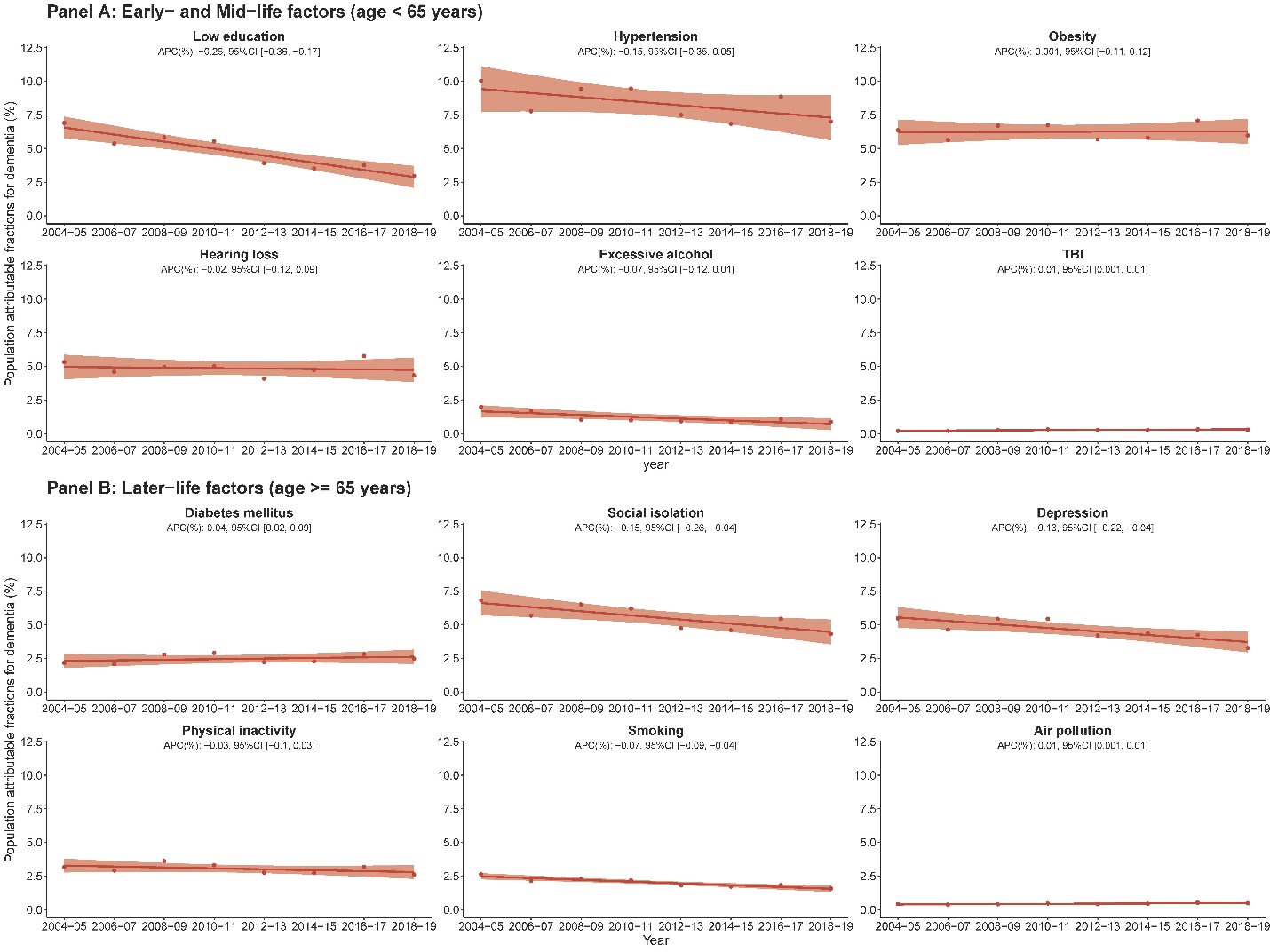


**Sup Figure 9. Temporal trends in population attributable fraction of 12 modifiable risk factors for dementia, by sex, excluding those with probable dementia or those responded by proxy during follow-up.** Average percentage change (APC) was used to quantify the temporal trend in population attributable fraction (PAF, as %), extracted from linear regression with PAF as the outcome and continuous form of year as the predictor. The APC indicates the extent to which the percentage points of PAF vary with each passing year.


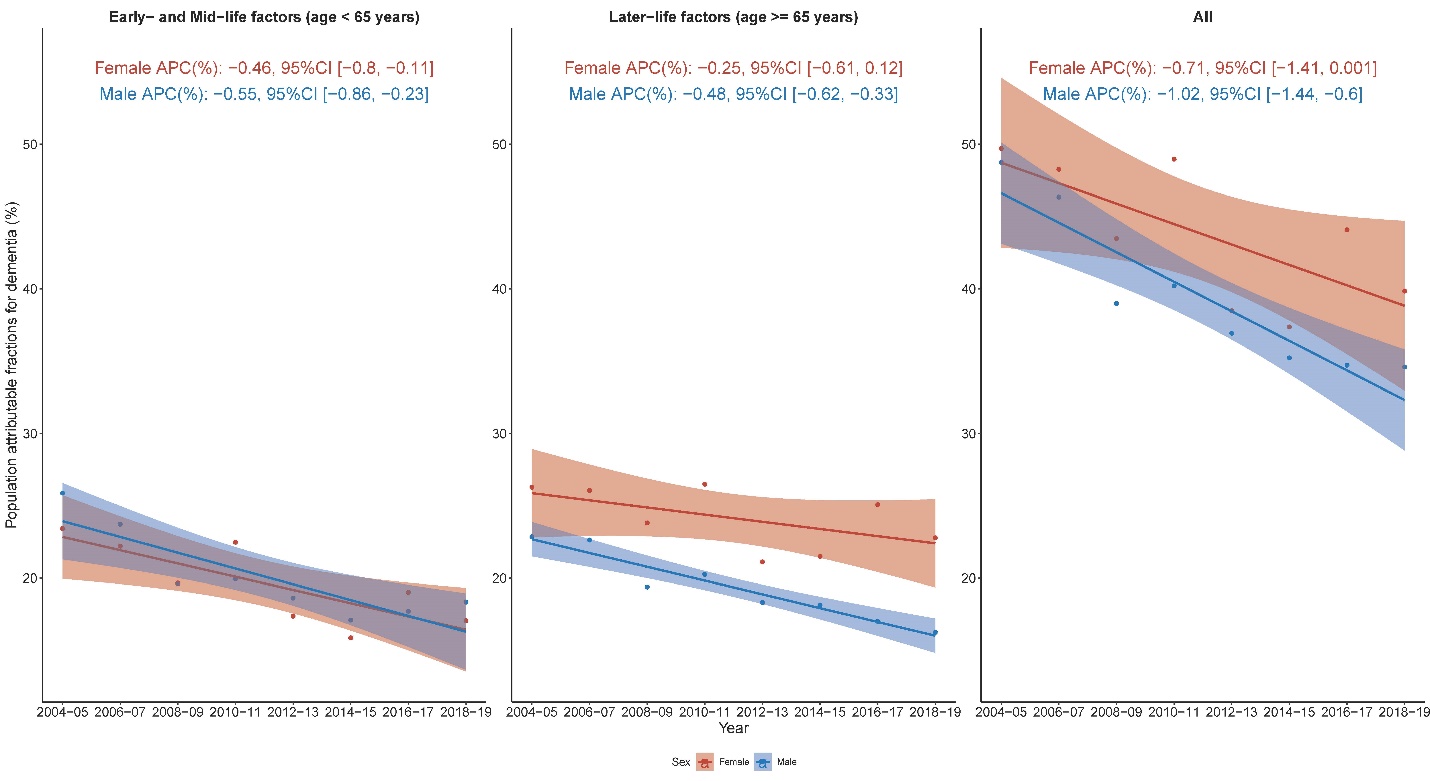


**Sup Figure 10. Temporal trends in population attributable fraction of 12 modifiable risk factors for dementia, by sex and risk factor, excluding those with probable dementia or those responded by proxy during follow-up.** Average percentage change (APC) was used to quantify the temporal trend in population attributable fraction (PAF, as %), extracted from linear regression with PAF as the outcome and continuous form of year as the predictor. The APC indicates the extent to which the percentage points of PAF vary with each passing year.


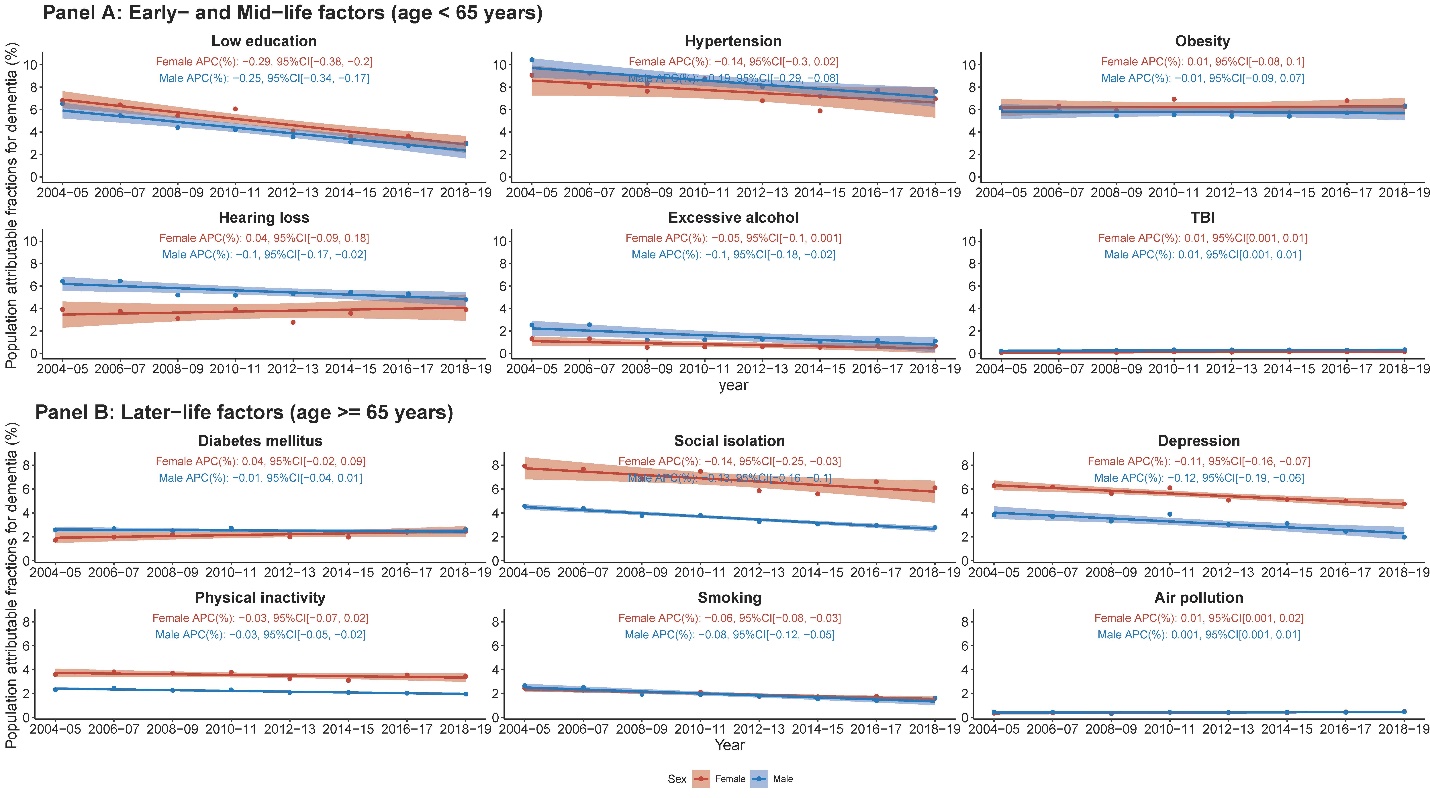


**Sup Figure 11. Temporal trends in population attributable fraction of 12 modifiable risk factors for dementia, by socioeconomic status, excluding those with probable dementia or those responded by proxy during follow-up.** Average percentage change (APC) was used to quantify the temporal trend in population attributable fraction (PAF, as %), extracted from linear regression with PAF as the outcome and continuous form of year as the predictor. The APC indicates the extent to which the percentage points of PAF vary with each passing year.


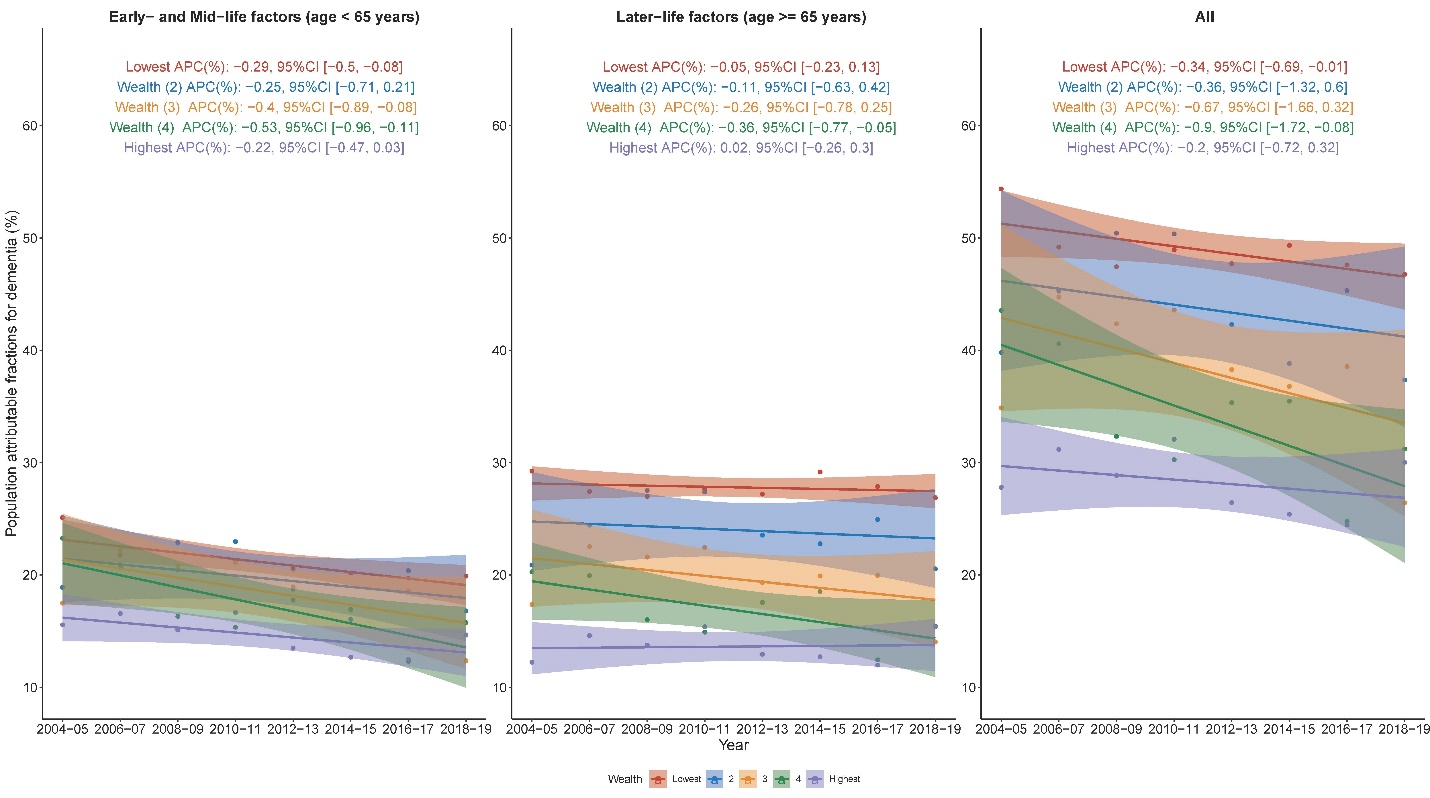


**Sup Figure 12. Temporal trends in population attributable fraction of 12 modifiable risk factors for dementia, by socioeconomic status and risk factor, excluding those with probable dementia or those responded by proxy during follow-up.** Average percentage change (APC) was used to quantify the temporal trend in population attributable fraction (PAF, as %), extracted from linear regression with PAF as the outcome and continuous form of year as the predictor. The APC indicates the extent to which the percentage points of PAF vary with each passing year.


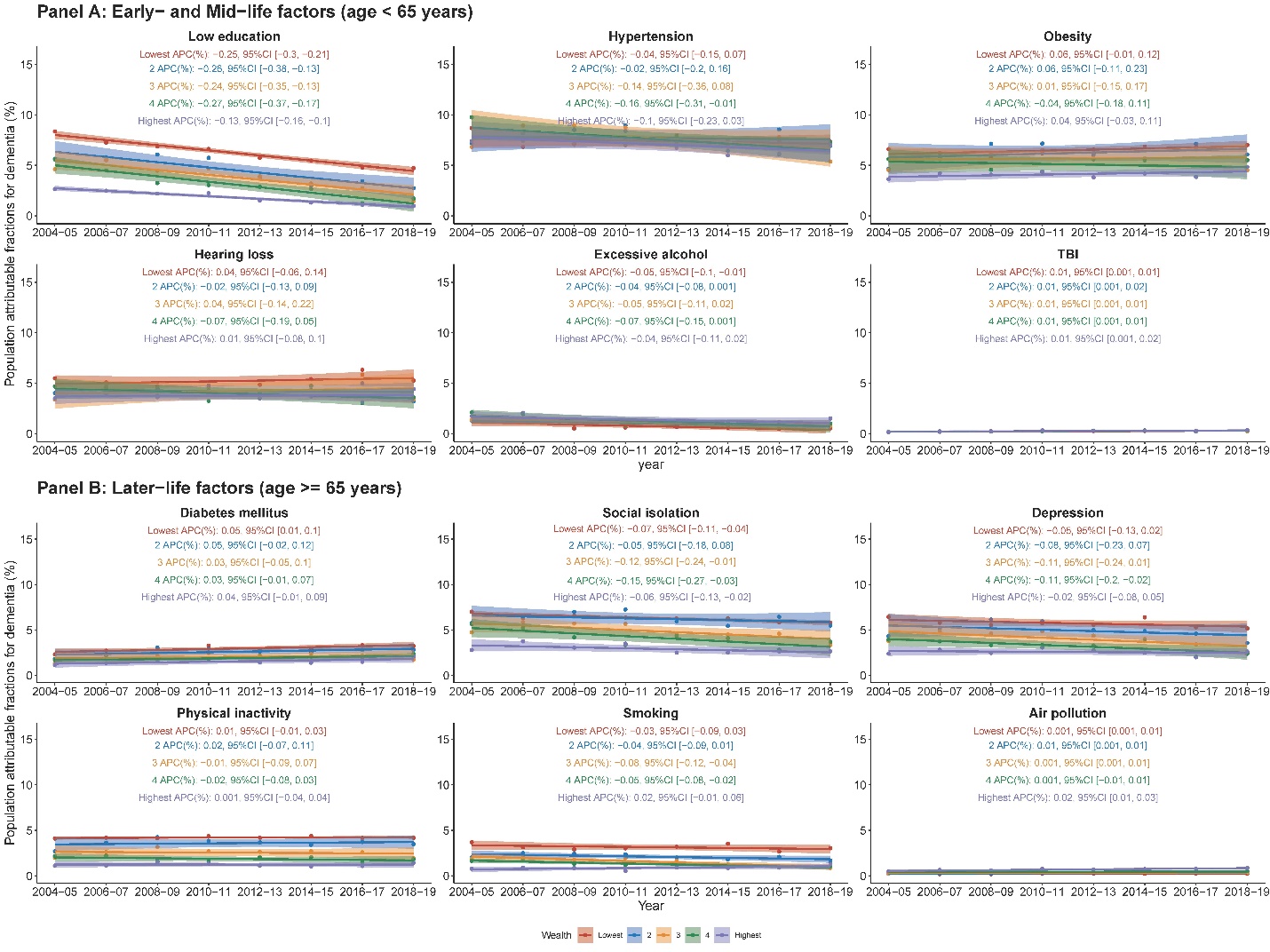

Supplement: Supplementary file 1 — Supplementary Material 1. [file 12916_2024_3464_MOESM1_ESM.docx]
